# LncRNA SEMA3B-AS1 inhibits breast cancer progression by targeting miR-3940/KLLN axis

**DOI:** 10.1038/s41419-022-05189-7

**Published:** 2022-09-19

**Authors:** Jin Hu, Haohao Huang, Zihan Xi, Shenghui Ma, Jie Ming, Fang Dong, Hui Guo, Huiqiong Zhang, Ende Zhao, Guojie Yao, Liu Yang, Feng Zhang, Wuping Zheng, Hengyu Chen, Tao Huang, Lei Li

**Affiliations:** 1 Department of Breast and Thyroid Surgery, Union Hospital, Tongji Medical College, Huazhong University of Science and Technology, Wuhan, 430022 China; 2 Department of Breast and Thyroid Surgery, Renmin Hospital of Wuhan University, Wuhan, China; 3 Department of Neurosurgery, General Hospital of Central Theater Command of Chinese People’s Liberation Army, Wuhan, 430070 PR China; 4 Department of Gastrointestinal Surgery, Union Hospital, Tongji Medical College, Huazhong University of Science and Technology, Wuhan, 430022 China; 5 Department of Emergency Medicine, Affiliated Hospital of Sergeant School Affiliated to Army Medical University, Shijiazhuang, 516562 China; 6 Department of Breast and Thyroid Surgery, The Second Affiliated Hospital of Hainan Medical University, Haikou, 570102 China

**Keywords:** Breast cancer, Cell growth

## Abstract

Long noncoding RNAs (lncRNAs) play crucial regulatory roles in the progression of various cancers. However, the functional roles of lncRNAs in breast cancer remain unclear. In this study, we investigated the functional role of a novel long noncoding RNA SEMA3B-AS1 (lncRNA SEAS1) in breast cancer progression and the underlying mechanisms. SEAS1 was downregulated in the triple-negative breast cancer (TNBC) tissues compared with the para-carcinoma tissues, which was associated with poor prognosis of TNBC patients. We demonstrated that SEAS1 knockdown significantly increased the proliferation, migration, and invasion of TNBC cell lines, whereas SEAS1 overexpression reversed these effects. Bioinformatics analysis demonstrated that microRNA (miR)-3940-3p was a potential target of SEAS1. Mechanistically, RNA immunoprecipitation (RIP) and luciferase reporter assays confirmed that lncRNA SEMA3B-AS1 acted as sponge for miR-3940-3p, preventing the degradation of its target gene KLLN, which acts as a tumor-inhibiter in TNBC. Moreover, RNA pulldown, mass spectrometry, ChIP, and luciferase reporter assays confirmed that SMAD3 directly interacted with the promoter of SEAS1 and suppressed its transcription, thereby promoting TNBC progression. The clinical samples of TNBC confirmed SEAS1 was correlated inversely with lymphatic and distant metastasis. In conclusion, our findings reveal a novel pathway for TNBC progression via SMAD3/lncRNA SEAS1/miR-3940-3p/KLLN axis, and suggest that SEAS1 may serve as a potential biomarker and therapeutic target for TNBC.

## Introduction

Breast cancer (BC) is the most common cancer type among females worldwide [1]. It is classified into three subtypes based on the molecular typing, namely, (1) luminal—estrogen receptor (ER) or progesterone receptor (PR) positive or both; (2) human epidermal growth factor receptor 2 (HER2) positive; and (3) triple-negative breast cancer (TNBC), which lack hormone receptor and HER2 expression [2–4].

TNBC accounts for 10–15% of all BC in the United States and is more common among African Americans and women <50 years of age [5]. In 2022, an estimated 290,560 new cases of BC and 43,780 BC-related deaths are expected in the United States [6]. Nearly 2.5 million cases of BC were expected in China by 2021 based on the population growth rates; TNBC accounts for nearly 20% of all the BC cases in China [7]. TNBC is associated with the worst prognosis and overall survival (OS) among all the subtypes of BC [8].

A previous study reported that more than 98% of the human genome does not encode proteins by genome sequencing technology [9]. Among these, transcripts that are longer than 200 nucleotides are defined as long non-coding RNAs (lncRNAs) [10, 11]. An increasing number of studies have shown that dysregulation of lncRNAs is closely related with cancer cell transformation, growth, apoptosis, metastasis, and chemotherapeutic resistance [12–15]. Many evidences suggests that during normal physiological and pathological conditions including tumorigenesis, lncRNAs act as competing endogenous RNAs (ceRNAs) that competitively bind and sequester their target miRNAs, thereby counteracting miRNA mediated repression of targeted mRNAs, indirectly regulating the expression of the miRNA target genes [16–20]. In this study, we investigated the role of lncRNA SEMA3B-AS1 (SEAS1) in breast cancer progression and the underlying mechanisms.

In present study, we revealed that the expression level of SEAS1 were significantly decreased in TNBC samples and SEAS1 served as a sponge for miR-3940-3p to attenuate its repressive effect on KLLN. Further exploration showed that SMAD3 inhibited the transcription activity of SEAS1 through binding to its promoter. This study describes a novel SMAD3–SEAS1–miR-3940-3p–KLLN axis and clarified the underlying regulatory mechanisms of SEAS1 in TNBC malignant progression, providing a potential diagnostic and therapeutic target for TNBC.

## Results

### LncRNA SEAS1 is downregulated in breast cancer tissues and cell lines

**Fig. 1:**
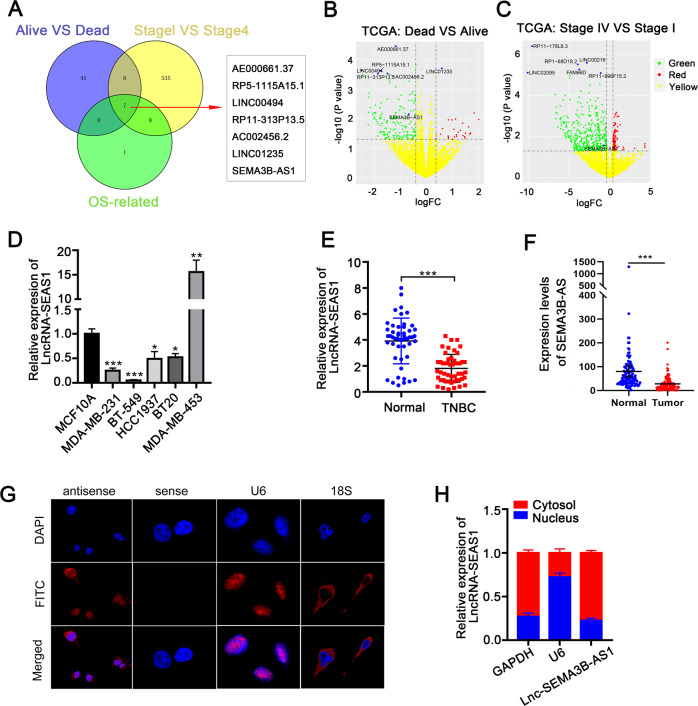
Characterization of lncRNA SEMA3B-AS1 as a novel tumor suppressor lncRNA in breast cancer. **A** LncRNA gene expression signatures from the TNBC cancer samples in the TCGA database were classified into three datasets based on their correlation with the survival status, tumor stage, and overall survival. Venn diagram shows the 7 candidate lncRNAs among the 3 datasets. **B**, **C** Volcano plots show the expression profiles of the 7-candidate prognostic lncRNAs in the Dead vs. Alive and Stage IV vs. Stage I datasets. **D** RT-qPCR analysis shows the expression of SEAS1 in various breast cancer cell lines and the normal breast epithelial cell line, MCF10A. GAPDH was used as the internal control. **E** RT-qPCR analysis shows the expression of lncRNA SEAS1 in 50 pairs of TNBC and adjacent normal breast tissues. GAPDH was used as the internal control. **F** The expression levels of SEAS1 based on TPM (transcript per million) in the breast cancer tissues and the normal breast tissues. **G** FISH assay results show the cytoplasmic localization of SEAS1 in the MDA-MB-231 cells. The nuclei were stained with DAPI (blue). **H** RT-qPCR analysis shows the expression levels of lncRNA SEAS1 in the nuclear and cytoplasmic fractions of the MDA-MB-231 cells. U6 and GAPDH were used as the nuclear and cytoplasmic markers, respectively. *P < 0.05, **P < 0.01, and ***P < 0.001. Representative data from at least 3 experiments with comparable results are shown.

The lncRNA-expression signatures from the TNBC samples in the Cancer Genome Atlas (TCGA) database were classified based on their correlation with the survival status, tumor stage, and overall survival. Then, analysis of the lncRNAs in these 3 datasets using the Venn diagram revealed 7 candidate lncRNAs (AE000661.37, RP5-115A15.1, LINC00494, RP11-313P13.5, AC002456.2, LINC01235, SEAS1) with potential prognostic value (Fig. 1A). The lncRNA expression profiles were analyzed using volcano plots in the Dead vs. Alive and Stage IV vs. Stage I datasets. Among the 7 prognostic-candidate lncRNAs, only SEAS1 was significantly down-regulated in the TNBC tissues compared with the normal breast tissues (Fig. 1B, C). The Gene Expression Profiling Interactive Analysis (GEPIA) database analysis of all the 7 candidate lncRNAs further demonstrated that only high expression levels of SEAS1 significantly correlated with favorable prognosis of breast cancer patients (Supplementary Fig. 1).

The localization and sequence of the full-length SEAS1 and its secondary structure based on minimum free energy (MFE) were shown in Supplementary Fig. 2A–C. The protein coding potential of SEAS1 was evaluated using the Open Reading Frame (ORF) Finder and the conserved domain database (https://lncipedia.org/). SEAS1 sequence did not show any putative protein coding potential according to the results of five different online protein-coding identification metrics (Supplementary Fig. 2D–F). Real-time quantitative PCR (RT-qPCR) assay showed that the expression levels of SEAS1 were significantly reduced in multiple TNBC cell lines compared to the normal breast epithelial cells (MCF10A) (Fig. 1D). RT-qPCR analysis also showed that SEAS1 was significantly downregulated in the TNBC tissues compared to the adjacent normal breast tissues (*n* = 50 pairs) (Fig. 1E). The expression levels of SEAS1 based on transcripts per million (TPM) in the breast cancer tissues were significantly lower than the normal breast tissues (Fig. 1F). Moreover, FISH analysis demonstrated that SEAS1 was localized in the cytoplasm of the MDA-MB-231 breast cancer cell line (Fig. 1G). The cytoplasmic localization of SEAS1 in the breast cancer cells was further confirmed by the nuclear/cytoplasmic RNA fractionation assay (Fig. 1H). Taken together, these results suggested that the lncRNA SEAS1 was downregulated in breast cancer and mainly localized in the cytoplasm of TNBC cells.

### SEAS1 regulates proliferation, apoptosis, and invasion of TNBC cells

**Fig. 2:**
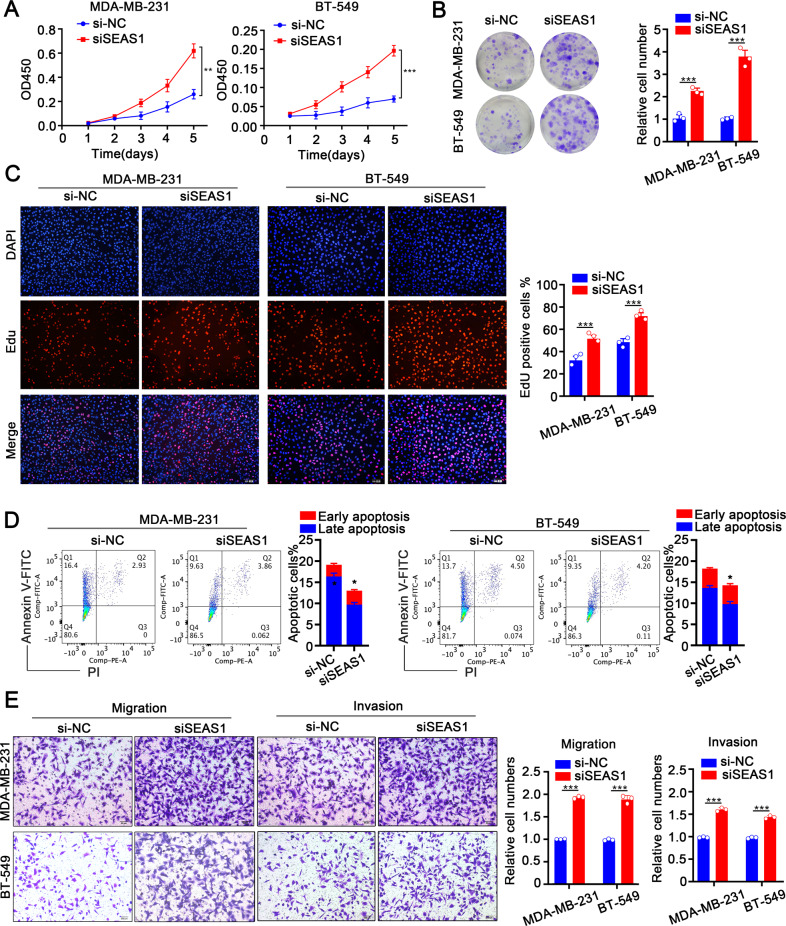
Low expression of SEAS1 facilitates proliferation, invasion, and inhibits apoptosis of TNBC cells. **A**–**C** CCK-8, colony formation, and EdU assay results show the proliferation rate status of control and SEAS1 silenced MDA-MB-231 and BT-549 cells. **D** Flow cytometry analysis shows the apoptotic rate in the control and SEAS1 knockdown MDA-MB-231 and BT-549 cells. The cells were stained with AnnexinV and PI. **E** Transwell assays show the migration and invasiveness of MDA-MB-231 and BT-549 cells transfected with si-NC or siSEAS1. The data represent average of three independent experiments. *P < 0.05, **P < 0.01, and ***P < 0.001. Representative data from at least 3 experiments with comparable results are shown.

To understand the roles of SEAS1 in TNBC progression, three kinds of siRNAs for human SEAS1 (siSEAS1) were transfected into MDA-MB-231 and BT-549 cells to knockdown the expression of SEAS1, and the knockdown efficiency was examined by RT-qPCR. The results showed that siSEAS1 transfection markedly reduced the expression of SEAS1 in MDA-MB-231 and BT-549 cells, so we selected siSEAS1#1 for further experiments (Supplementary Fig. 3A). CCK8, colony formation, and EdU assay results demonstrated that the proliferation of the SEAS1-knockdown MDA-MB-231 and BT-549 cells was significantly higher compared to the control groups (Fig. 2A–C). Further flow cytometry analysis suggested that SEAS1 silencing conspicuously repressed cell apoptosis in TNBC cells (Fig. 2D). The results of transwell assay indicated that SEAS1 silencing exhibited more aggressive migratory and invasive potential in MDA-MB-231 and BT-549 cells (Fig. 2E). These results demonstrated that SEAS1 regulated proliferation, survival, and progression of the TNBC cells.

**Fig. 3:**
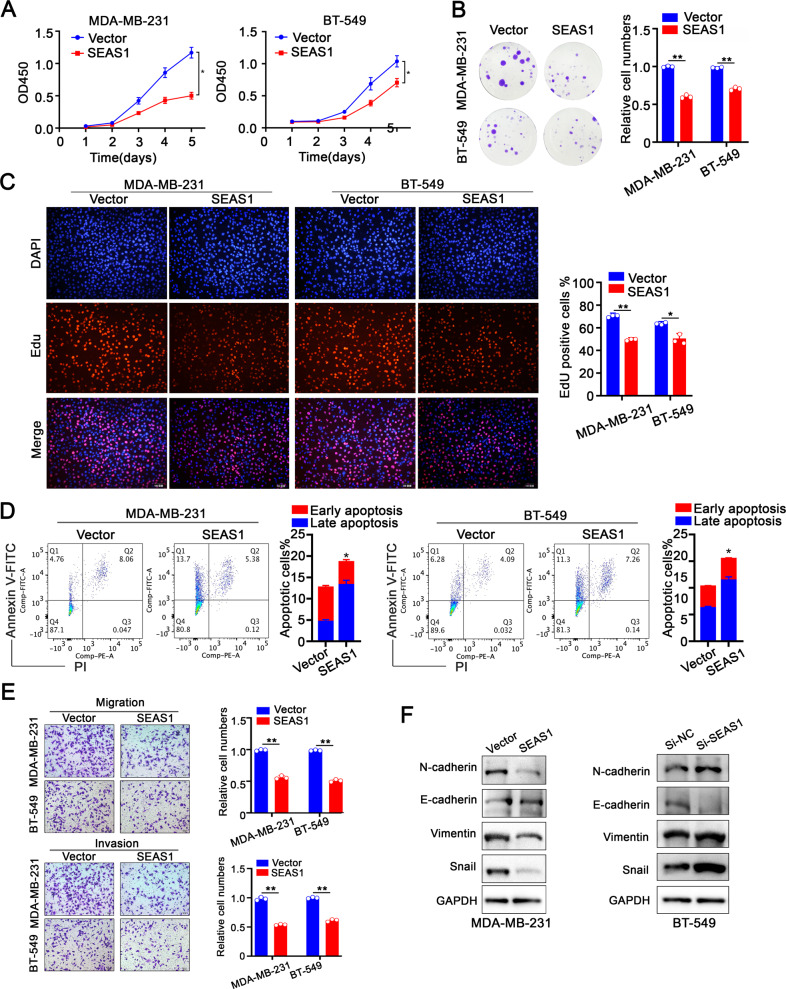
SEAS1 overexpression suppresses proliferation, invasion, and facilitates apoptosis of TNBC cells. **A**–**C** CCK8, colony formation, and EdU assay results show the proliferation rates of control and SEAS1 overexpressing MDA-MB-231 and BT-549 cells. **D** Flow cytometry results show the apoptotic rates in the control and SEAS1 overexpressing MDA-MB-231 and BT-549 cells. **E** Transwell assays show the migration and invasion rates of control vector-transfected and SEAS1-overexpression vector-transfected MDA-MB-231 and BT-549 cells. The data represent average of three independent experiments. **F** Western blot analysis shows the relative expression levels of EMT-related proteins, namely, N-cadherin, Vimentin, Snail, and E-cadherin in the control, SEAS1-silenced, and SEAS1-overexpressing MDA-MB-231 or BT-549 cells. *P < 0.05, **P < 0.01, and ***P < 0.001. Representative data from at least 3 experiments with comparable results are shown.

We further confirmed the tumor suppressor function of SEAS1 in the TNBC cells by transfecting MDA-MB-231 and BT-549 cells with a lentiviral vector containing the SEAS1 sequence or with the lentiviral vector to generate vector control group cells (Vector) (Supplementary Fig. 3B). SEAS1 overexpression significantly reduced the proliferation ability of the MDA-MB-231 and BT-549 cells (Fig. 3A–C). FACS analysis by Annexin V/PI co-staining demonstrated that the apoptotic rate was dramatically higher in the SEAS1-overexpressing MDA-MB-231 and BT-549 cells compared to the vector-transfected control groups cells (Fig. 3D). Furthermore, SEAS1 overexpression significantly reduces the in-vitro migration and invasion of the MDA-MB-231 and BT-549 cells (Fig. 3E). Given SEAS1 affected the proliferation, apoptosis, especially migration and invasion of TNBC cells, we further detected the expression levels of EMT-related proteins in the SEAS1-overexpressing and the SEAS1-silenced TNBC cells. In contrast with the SEAS1-silenced MDA-MB-231 and BT-549 cells, the SEAS1-overexpressing showed distinct downregulation of the mesenchymal marker proteins such as N-cadherin, Vimentin, and Snail, and significantly upregulated the expression of the epithelial marker protein E-cadherin (Fig. 3F). Overall, the above results imply SEAS1 might serve as an inhibiter in TNBC progression.

### SEAS1 acts as a sponge for miR-3940-3p in the TNBC cells

**Fig. 4:**
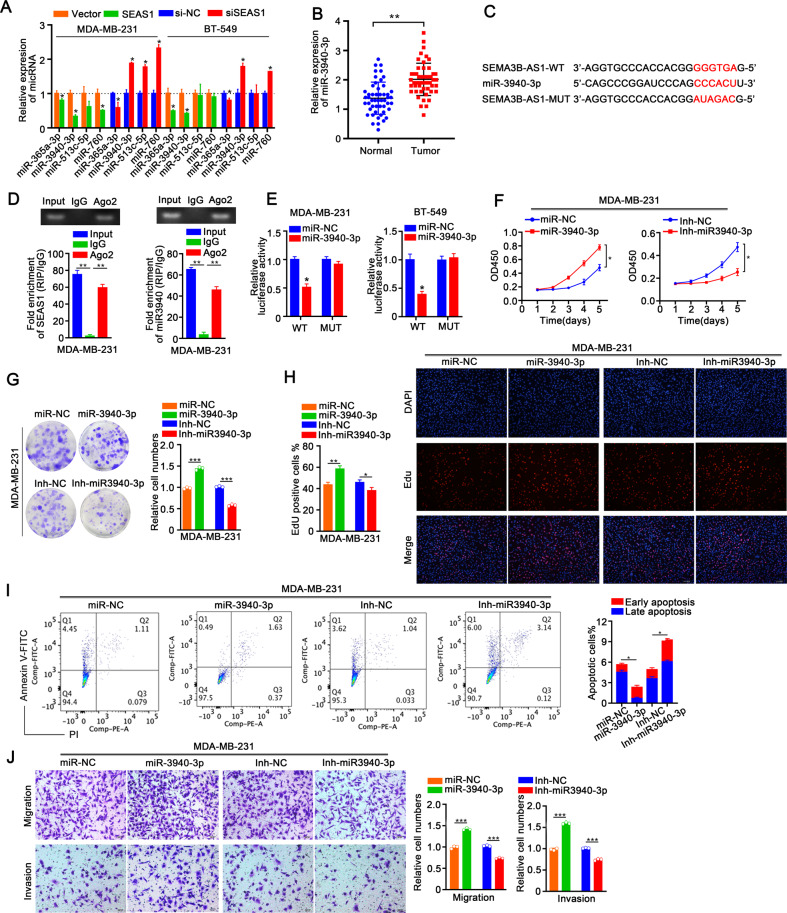
LncRNA SEAS1 sponges miR-3940-3p in the TNBC cells. **A** RT-qPCR analysis shows the levels of hsa-miR-3940-3p in the control, SEAS1-silenced, and SEAS1-overexpressing MDA-MB-231 and BT-549 cells. **B** RT-qPCR analysis shows the expression levels of miR-3940-3p in the breast cancer tissues and normal breast tissues (*n* = 50). GAPDH was used as the internal control. **C** Schematic diagram shows the predicted wild-type and mutated binding sites for miR-3940-3p in SEAS1. **D** RT-qPCR analysis shows the levels of SEAS1 or miR-3940-3p pulled down from the lysates of MDA-MB-231cells with the anti-AGO2 antibody in the RIP assay. **E** Luciferase activity of the reporter construct containing the wild-type or miR-3940 binding mutant of SEAS1 was measured after cotransfection of the reporter with Negative Control (NC) or miR-3940-3p in MDA-MB-231 and BT-549 cells. **F**–**H** CCK8, colony formation, and EdU assays show the proliferation rates of the control, miR-3940-3p overexpressing and miR-3940-3p-depleted MDA-MB-231 cells. **I** Flow cytometry analysis shows the proportion of apoptotic cells in the control, miR-3940-3p overexpressing and miR-3940-3p-depleted MDA-MB-231 cells. **J** Transwell assays show the migration and invasion rates of the control, miR-3940-3p overexpressing and miR-3940-3p-depleted MDA-MB-231 cells. The data show an average of three independent experiments. *P < 0.05, **P < 0.01, and ***P < 0.001. Representative data from at least 3 experiments with comparable results are shown.

LncRNAs mainly function as ceRNAs and regulate the expression of their target genes including oncogenes by binding and sequestering their target miRNAs [21, 22]. Since SEAS1 is mainly localized in the cytoplasm, we hypothesized that it may act as a sponge for specific miRNAs and regulate their binding to the target mRNAs. We identified four candidate miRNAs, including hsa-miR-3940-3p (miR-3940-3p) by screening the potential target miRNAs of SEAS1 using the online miRNA search software in the DIANA Lab Tools website (Carolina.imis.athena-innovation.gr/diana_tools/). RT-qPCR analysis of the candidate miRNAs in the SEAS1-silenced and SEAS1-overexpressing MDA-MB-231 and BT-549 cells showed that miR-3940-3p was the SEAS1 target miRNA (Fig. 4A). Furthermore, we assessed the levels of pri-miR-3940-3p and pre-miR-3940-3p in the control and SEAS1-silenced TNBC cells. RT-qPCR analysis showed that SEAS1 knockdown did not change the levels of both pri-miR-3940-3p and pre-miR-3940-3p in the TNBC cells (Supplementary Fig. 3C and D). This suggested that SEAS1 regulated the expression of miR-3940-3p in the TNBC cells at the post-transcription level. Moreover, we further investigated the expression levels of miR-3940-3p in 50 pairs of TNBC and normal breast samples using RT-qPCR analysis, which showed that miR-3940-3p was significantly up-regulated in breast cancer tissues compared to adjacent normal tissues (Fig. 4B). In addition, the potential binding sites of miR-3940-3p in the SEAS1 sequence were shown in Fig. 4C.

During RNA silencing, mature miRNAs in concert with the RNA-induced silencing complexes (RISCs) act as a template for recognizing complementary mRNAs. Ago2 protein is an essential component of RISC. Therefore, we performed RNA immunoprecipitation (RIP) with the anti-Ago2 antibody to verify the interactions between SEAS1 and Ago2 as well as miR-3940-3p and Ago2. RIP assay results showed that both SEAS1 and miR-3940-3p were pulled down by the anti-Ago2 antibody (Fig. 4D). This suggested potential binding between SEAS1 and miR-3940-3p. RIP assay also confirmed the interaction between SEAS1 and miR-3940-3p in the xenograft tumor tissues (Supplementary Fig. 3E). To further demonstrate that the alteration of luciferase activity induced by SEAS1, we cloned SEAS1 with the predicted wild-type miR-3940-3p binding site (wild-type, WT) or with the mutated miR-3940-3p binding site (mutant type, MUT) into the luciferase report vector. The WT and MUT luciferase report vectors were co-transfected with miR-3940-3p mimics into the MDA-MB-231 and BT-549 cells. Relative firefly luciferase activity was significantly reduced in MDA-MB-231 and BT-549 cells transfected with the SEAS1-WT luciferase reporter and miR-3940-3p mimics, but the relative firefly luciferase activity was not reduced in the MDA-MB-231 and BT-549 cells transfected with the SEAS1-MUT luciferase and miR-3940-3p mimics (Fig. 4E). These data confirmed direct binding of miR-3940-3p by SEAS1.

### MiR-3940-3p affects proliferation, apoptosis, and progression of the TNBC cells

We then examined the role of miR-3940-3p in the breast cancer cells. After miR-3940-3p overexpression, CCK8, colony formation, EdU, and Annexin V/PI flow cytometry assays showed that the cell viability was enhanced as well as a decreased apoptotic rate in MDA-MB-231 and BT-549 cells, whereas inhibited miR-3940-3p exhibited the opposite results (Fig. 4F–I, Supplementary Fig. 4A–D). Coincident with these results, the transwell assay confirmed that miR-3940-3p overexpression facilitated the migration and invasion of TNBC cells, which were suppressed when miR-3940-3p was silenced (Fig. 4J, Supplementary Fig. 4E). Therefore, these data suggested that miR-3940-3p, which was the target of lncRNA SEAS1, accelerated TNBC cells growth and metastasis progression.

### SEAS1 inhibits TNBC progression by targeting miR-3940-3p

**Fig. 5:**
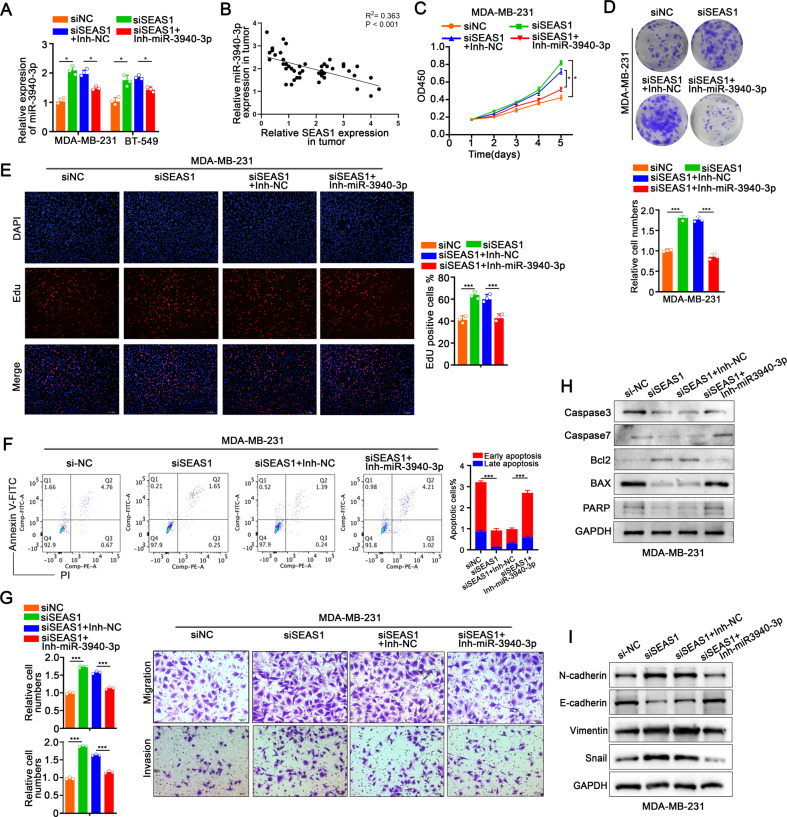
SEAS1 inhibits TNBC progression by targeting miR-3940-3p. **A** RT-qPCR results show the expression levels of miR-3940-3p in the MDA-MB-231 cells co-transfected with siRNA against SEAS1 and the miR-3940-3p inhibitor. **B** Spearman correlation analysis results show the relationship between miR-3940-3p and SEAS1 expression levels in 50 breast tumor tissues. **C**–**E** CCK8, colony formation, and EdU assay results show the proliferation rates of MDA-MB-231 cells co-transfected with siRNA against SEAS1 and the miR-3940-3p inhibitor. **F** FACS analysis results show the apoptotic rates in the MDA-MB-231 cells co-transfected with siRNA against SEAS1 and the miR-3940-3p inhibitor and the corresponding controls. **G** Transwell assay results show the migration and invasion rates of MDA-MB-231 cells co-transfected with siRNA against SEAS1 and miR-3940-3p inhibitor and the corresponding controls. **H** Western blot assay shows the levels of caspase 3, caspase 7, BAX, PARP, and Bcl2 proteins in the MDA-MB-231 cells co-transfected with siRNA against SEAS1 and the miR-3940-3p inhibitor and the corresponding controls. **I** Western blot analysis shows the levels of EMT-related proteins namely, N-cadherin, Vimentin, Snail, and E-cadherin in MDA-MB-231 cells co-transfected with siRNA against SEAS1 and the miR-3940-3p inhibitor and the corresponding controls. *P < 0.05, **P < 0.01, and ***P < 0.001. Representative data from at least 3 experiments with comparable results are shown.

MDA-MB-231 and BT-549 cells were transfected with SEAS1-siRNA alone or SEAS1-siRNA combined with miR-3940-3p inhibitor to determine whether SEAS1 regulated TNBC cell progression by targeting miR-3940-3p. SEAS1 silencing increased the levels of miR-3940-3p, but this effect was abrogated by treatment with the miR-3940-3p inhibitor (Fig. 5A). Spearman correlation analysis demonstrated that miR-3940-3p expression negatively correlated with the levels of SEAS1 in the breast tumor specimens (Fig. 5B). SEAS1 silencing markedly increased proliferation, migration, invasiveness as well as suppressed apoptosis, but these effects were reversed in the MDA-MB-231 and BT-549 cells that co-transfected with siSEAS1 and miR-3940-3p inhibitor (Fig. 5C–G, Supplementary Fig. 5A–E). Western blot results further demonstrated that SEAS1 silencing significantly decreased the levels of pro-apoptotic proteins, caspase 3, caspase 7, BAX, and PARP, and increased the levels of the anti-apoptotic protein, Bcl2, in MDA-MB-231 cells, but these effects were reversed in the MDA-MB-231 cells co-transfected with siSEAS1 and miR-3940-3p inhibitor (Fig. 5H). Furthermore, SEAS1-silenced TNBC cells showed significant upregulation of the levels of mesenchymal marker proteins such as N-cadherin, Vimentin, and Snail, and significant downregulation of the levels of epithelial marker protein, E-cadherin, but these effects were reversed in the TNBC cells co-transfected with siSEAS1 plus the miR-3940-3p inhibitor (Fig. 5I, Supplementary Fig. S5F). Taken together, these data confirmed that SEAS1 inhibited TNBC progression by targeting miR-3940-3p.

### KLLN is the direct target gene of miR‑3940‑3p

**Fig. 6:**
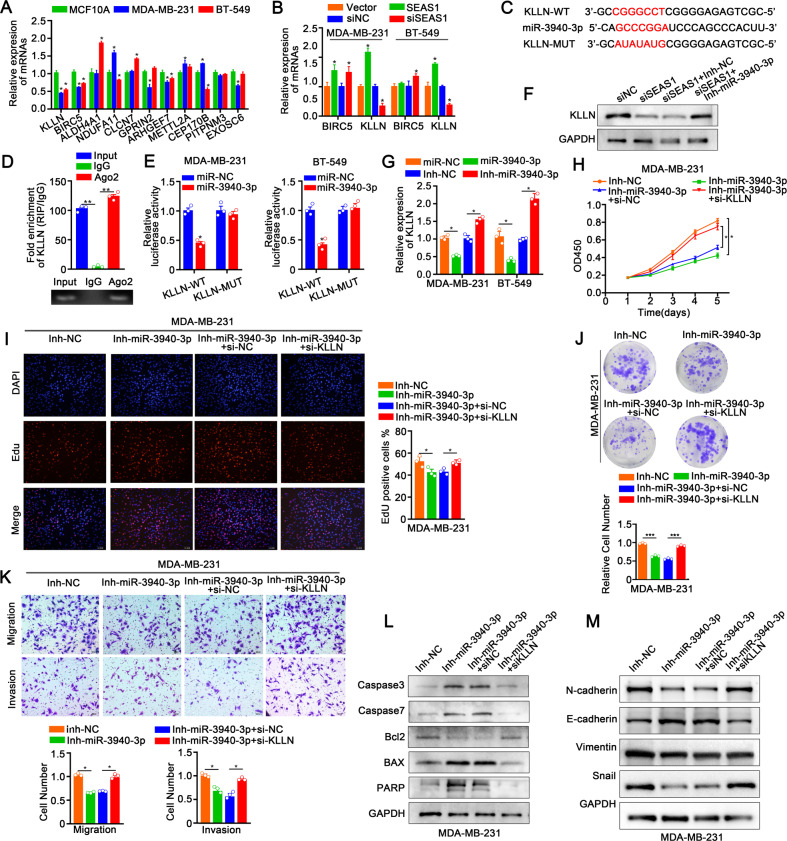
KLLN is the direct target gene of miR‑3940‑3p. **A** RT-qPCR analysis shows the expression levels of several miR-3940-3p target genes including KLLN in the TNBC cell lines and the normal breast epithelial cell line. **B** RT-qPCR analysis shows the expression levels of the miR-3940-3p target genes, BIRC5 and KLLN in the control, SEAS1-silenced, and SEAS1-overexpressing TNBC cell lines. **C** The wild-type and mutated binding sites of miR-3940-3p in the 3′-UTR of KLLN. **D** RT-qPCR analysis shows the results of anti-AGO2 RIP assay. The relative levels of KLLN mRNA pulled down from the MDA-MB-231cell extracts using anti-Ago2 antibody and IgG are shown with the input. **E** Luciferase reporter assays show the luciferase activity in MDA-MB-231 cells co-transfected with luciferase reporter vector cloned with the wild-type or mutant 3′UTR sequence of KLLN. **F** Western blot analysis shows the levels of KLLN protein in SEAS1-silenced TNBC cells co-transfected with miR-3940-3p inhibitor and the corresponding controls. **G** RT-qPCR analysis shows the levels of KLLN mRNA in SEAS1-silenced TNBC cells transfected with the miR-3940-3p inhibitor and the corresponding controls. **H**–**J** CCK8, colony formation, and EdU assay results show the proliferation rates of the TNBC cells co-transfected with miR-3940-3p inhibitor and siRNA against KLLN (si-KLLN) and the corresponding controls. **K** Transwell assay results show the migration rates of TNBC cells co-transfected with the miR-3940-3p inhibitor and si-KLLN and the corresponding controls. **L** Western blot results show the levels of caspase 3, caspase 7, BAX, PARP, and Bcl2 proteins in the TNBC cells co-transfected with the miR-3940-3p inhibitor and si-KLLN and the corresponding controls. **M** Western blot results show the levels of EMT-related proteins, namely, N-cadherin, Vimentin, Snail, and E-cadherin in TNBC cells co-transfected with the miR-3940-3p inhibitor and si-KLLN and the corresponding controls. *P < 0.05, **P < 0.01, and ***P < 0.001. Representative data from at least 3 experiments with comparable results is shown in the figure.

Next, we identified 11 potential target genes of miR-3940-3p by analyzing online databases such as miRDB (http://mirdb.org/), miRPathDB (https://mpd.bioinf.uni-sb.de/), miRBase (https://mirbase.org/), and TargetScan (http://www.targetscan.org/). Among these 11 potential miR-3940-3p target genes, only BIRC5 and KLLN were down-regulated in the MDA-MB-231 and BT-549 cells compared with the control MCF10A normal breast cells (Fig. 6A). KLLN expression levels were significantly elevated in SEAS1-overexpressing TNBC cells and distinctly decreased after silence of SEAS1. However, this result was not seen for BIRC5, so we selected KLLN for further investigation (Fig. 6B). After systematic bioinformatics prediction via miRWalk database, we found that KLLN mRNA had a binding site for miR-3940-3p in its 3′UTR (Fig. 6C). Immunoprecipitation assays with the anti-Ago2 antibody pulled down endogenous KLLN mRNA from the MDA-MB-231 cell lysates (Fig. 6D). We then performed the dual-luciferase reporter assay to verify whether miR-3940-3p directly targeted the 3′UTR of KLLN. Relative firefly luciferase activity was significantly reduced in MDA-MB-231 and BT-549 cells co-transfected with the KLLN-WT-3′UTR plasmid and miR-3940-3p mimics, but the relative firefly luciferase activity was not affected in the MDA-MB-231 and BT-549 cells co-transfected with the KLLN-MUT-3′UTR plasmid and miR-3940-3p mimics (Fig. 6E). Western blot showed that SEAS1 silencing significantly decreased the levels of KLLN in the MDA-MB-231 cells, but these effects were abrogated in the MDA-MB-231 cells co-transfected with siSEAS1 and miR-3940-3p inhibitor (Fig. 6F). RT-qPCR results also showed negative association between KLLN mRNA and miR-3940-3p levels in the MDA-MB-231 and BT-549 cells (Fig. 6G). Collectively, these results suggested that KLLN was a direct target gene of miR‑3940‑3p in the TNBC cells.

### KLLN silencing rescue the inhibited effects of miR‑3940-3p

Based on the results mentioned above, we speculated miR-3940-3p could participate in regulating TNBC malignant progress by interacting with KLLN. The vitro rescue experiment was conducted to evaluate the regulatory role that KLLN plays in miR-3940-3p mediated TNBC progression. Downregulation of miR-3940-3p decreased proliferation, migration, and invasiveness of the MDA-MB-231 and BT-549 cells, but these effects were reversed by KLLN silencing (Fig. 6H–K, Supplementary Fig. 6A–D). As demonstrated by western blot assay, miR-3940-3p inhibition facilitated cell apoptosis in MDA-MB-231 and BT-549 cells, whereas suppression of KLLN restored this effect (Fig. 6L). Furthermore, western blot demonstrated that TNBC cells co-transfected with the miR-3940-3p inhibitor and si-KLLN promoted EMT, but these effects were reversed by the miR-3940-3p inhibitor (Fig. 6M, Supplementary Fig. 6E). These results demonstrated that miR-3940-3p promoted TNBC progression by silencing KLLN.

### SMAD3/SEAS1/miR-3940-3p/KLLN axis regulates TNBC progression

**Fig. 7:**
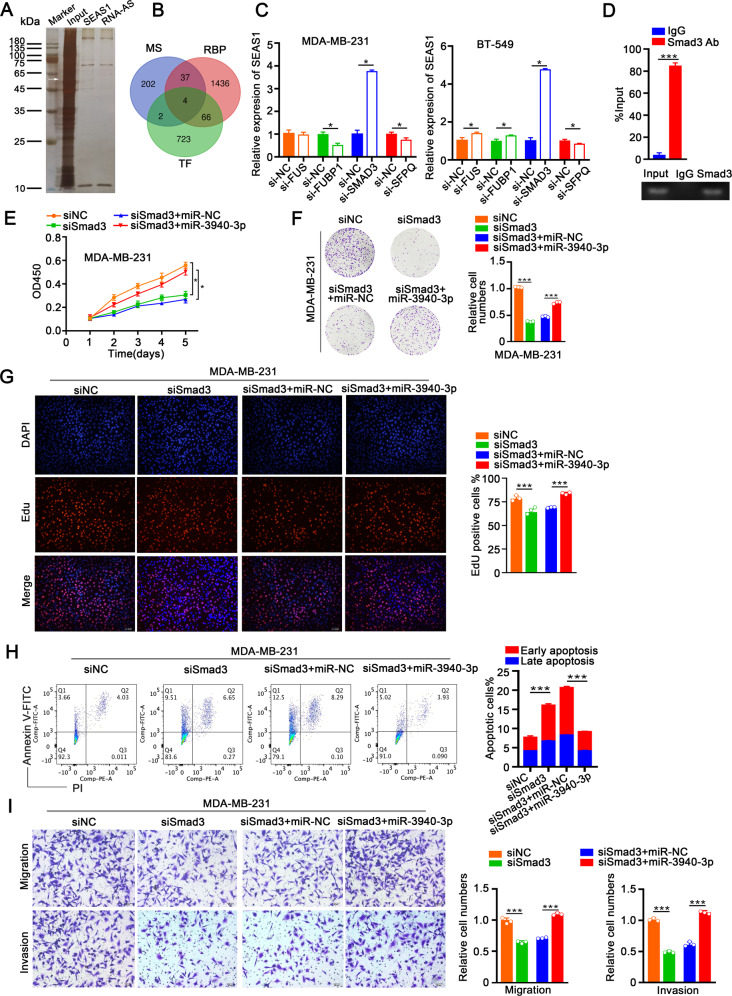
SMAD3/SEAS1/miR-3940-3p/KLLN axis regulates TNBC progression. **A** Silver stained SDS-PAGE blot shows the total proteins from the MDA-MB-231 cell lysates that are pulled down by the biotin-labeled SEAS1 in the biotinylated RNA pull-down assay. **B** Venn diagram shows the overlapping genes based on the intersection between the mass spectrometry data, RNA binding proteins, and transcription factors. **C** RT-qPCR results show the relative levels of SEAS1 in TNBC cells transfected with siRNAs against 4 potential SEMA3B-AS1-binding transcription factors, namely, FUS, FUBP1, SMAD3, and SFPQ. **D** RT-qPCR analysis shows SEAS1 levels that are pulled down in ChIP assay with the anti-SMAD3 antibody or IgG. MDA-MB-231cell lysates were used for the ChIP assay. **E**–**G** CCK-8, colony formation, and EdU assay results show the proliferation rates of MDA-MB-231cells co-transfected with siSMAD3 and miR-3940-3p mimic and the corresponding controls. **H** Flow cytometry analysis shows the apoptotic rate in the MDA-MB-231cells co-transfected with siSMAD3 and miR-3940-3p mimic and the corresponding controls. **I** Transwell assay results show the migration and invasion rates of MDA-MB-231cells co-transfected with siSMAD3 and miR-3940-3p mimic and the corresponding controls. *P < 0.05, **P < 0.01, and ***P < 0.001. Representative data from at least 3 experiments with comparable results are shown.

Several studies have shown that lncRNAs regulate cellular processes and pathways by interacting with RNA binding proteins. Therefore, to identify the RNA binding proteins interacting with lncRNA SEAS1, we performed RNA pull-down assay of the cytoplasmic extracts of MDA-MB-231 cells using the biotin-labeled SEAS1 transcript. The captured proteins were analyzed by silver staining and a strong band was evident at ~60 kDa compared to the negative control (Fig. 7A). We then evaluated mass spectrometry data of this band at ~60 kDa with the transcription factors (TF) and RNA-binding protein datasets and identified four potential candidate TFs, namely, FUS, FUBP1, SMAD3, and SFPQ (Fig. 7B). Then, the expression levels of SEAS1 were analyzed after silencing FUS, FUBP1, SMAD3, and SFPQ in the MDA-MB-231 and BT-549 cells. RT-qPCR assay results showed that the expression levels of SEAS1 were significantly increased in the SMAD3-silenced MDA-MB-231 and BT-549 cells, but the levels of SEAS1 were not affected by silencing FUS, FUBP1, and SFPQ (Fig. 7C). PROMO database analysis predicted the potential SMAD3 binding site in the SEAS1 promoter (Supplementary Fig. 3F). This interaction between SMAD3 and SEAS1 promoter was further confirmed by the ChIP assay with the lysates of MDA-MB-231 cells. ChIP assay results showed that SEAS1 was significantly enriched with the anti-SMAD3 antibody compared to the control IgG (Fig. 7D). The regulatory effect of SMAD3 on SEAS1 was validated using the luciferase reporter assay. The relative luciferase activity was reduced in TNBC cells co-transfected with the pGL3 reporter vector cloned with the wild-type SEAS1 promoter (SEAS1-WT) and SMAD3 overexpression vector (Supplementary Fig. 3G), but was increased in TNBC cells co-transfected with SEAS1-WT and siSMAD3 (Supplementary Fig. 3H). Furthermore, luciferase activity of the TNBC cells transfected with the pGL3 reporter vector cloned with the mutant-type SEAS1 promoter (SEAS1-MUT) was similar to the negative control (vector) group in the presence of SMAD3 overexpression vector as well as siSMAD3.

Next, we analyzed the effects of SMAD3 on the miR-3940-3p/KLLN axis by estimating the expression of miR-3940-3p and KLLN in SMAD3-silenced and SMAD3-overexpressing TNBC cells. KLLN levels were negatively regulated by SMAD3 in the TNBC cells (Supplementary Fig. 7A–B). Furthermore, SMAD3 silencing significantly reduced the proliferation, migration, and invasion of the TNBC cells, but these effects were reversed by silencing SEAS1 (Supplementary Fig. 7C–F).

Therefore, ablation of SMAD3 phenocopied the overexpression of SEAS1 by inhibiting proliferation, migration, invasion, and accelerated apoptosis of the TNBC cells, but these effects were rescued by ectopic expression of miR-3940-3p in the MDA-MB-231 and BT-549 cells (Fig. 7E–I, Supplementary Fig. 8A–E). These data demonstrated that SEAS1 expression was regulated by SMAD3 in the TNBC cells.

### SEAS1 overexpression inhibits tumor progression and highly correlated with favorable prognosis of TNBC

**Fig. 8:**
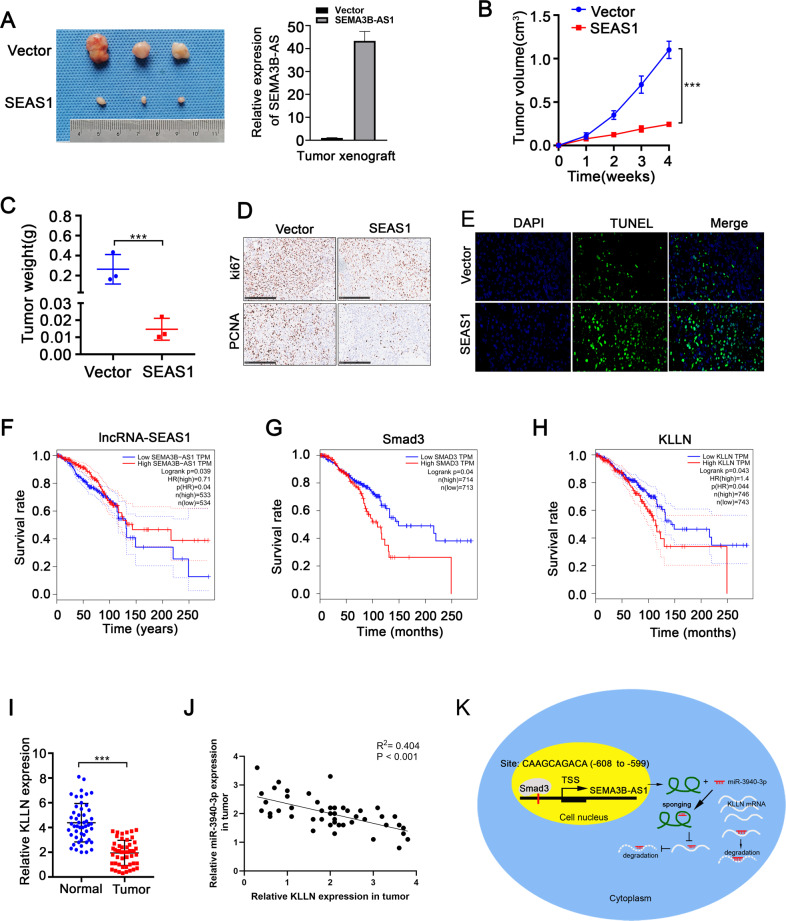
SEAS1 overexpression inhibits tumor progression and highly correlated with favorable prognosis of TNBC. **A** Representative images of xenograft tumors derived from SEAS1-overexpressing and vector control MDA-MB-231cells that were subcutaneously injected into Balb/c athymic nude mice. Relative expression levels of SEAS1 in the SEAS1-overexpressing and vector control MDA-MB-231cells are also shown. **B**, **C** Tumor weights and tumor volumes of xenograft tumors derived from the SEAS1-overexpressing and vector control MDA-MB-231cells are shown. Tumor volumes were calculated as volume = length × (width)^2^/2. The data are represented as mean ± SD. **D** Representative images show the differences between Ki67 and PCNA staining in the xenograft tumor tissues derived from SEAS1-overexpressing and vector control MDA-MB-231cells. **E** TUNEL assay results show the percentage apoptotic cells in the xenograft tumor tissues derived from SEAS1-overexpressing and vector control MDA-MB-231cells. **F–H** Kaplan–Meier Plotter database analysis shows the survival rate of breast cancer patients in the GEPIA dataset with high and low expression levels of **F** SEAS1, **G** SMAD3, and **H** KLLN. **I** RT-qPCR analysis shows the expression levels of KLLN mRNA in TNBC tissues and the adjacent normal breast tissues. **J** Spearman correlation analysis shows significant negative correlation between miR-3940-3p and KLLN expression levels in the breast cancer tissues (*R*^2^ = 0.527, *P* = 0.017). **K** Diagrammatic representation of the regulation of TNBC progression by the SMAD3/SEAS1/miR-3940-3p/KLLN axis. SMAD3 regulates transcription of SEAS1, which functions as a sponge for miR-3940-3p, the negative regulator of KLLN. *P < 0.05, **P < 0.01, and ***P < 0.001. Representative data from at least 3 experiments with comparable results are shown.

Next, we generated a subcutaneous xenograft tumor model in BALB/c athymic nude mice to validate the in vivo biological function of lncRNA SEAS1. MDA-MB-231 cells were stably transfected with the lentiviral vector containing either the negative control or SEAS1. The transformed cells (negative control and SEAS1-overexpressing MDA-MB-231 cells) were subcutaneously injected into the BALB/c athymic nude mice. The xenograft tumor weight and volumes derived from SEAS1 overexpressing TNBC cells were significantly lower compared to those derived from the negative control cells (Fig. 8A–C). Immunohistochemistry assays showed that SEAS1 overexpression decreased Ki67 and PCNA expression in the xenograft tumors (Fig. 8D). This suggested that SEAS1 overexpression suppressed the in vivo proliferation of the xenografted TNBC cells. TUNEL assay results demonstrated increased apoptosis in the xenograft tumors derived from the SEAS1 overexpressing TNBC cells compared to the negative control (Fig. 8E).

Next, we verified the clinical significance of SEAS1 by analyzing the correlation between SEAS1 expression levels and the clinicopathologic features of the TNBC patients. SEAS1 expression showed negative correlation with lymph node invasion and distant metastasis in the TNBC patients (Supplementary Table 4). GEPIA database analysis showed that higher expression levels of SEAS1 correlated with favorable prognosis and higher overall survival of the TNBC patients (Fig. 8F). Subsequently, we analyzed overall survival rates of TNBC patients based on SMAD3 and KLLN expression levels. Higher expression of SMAD3 correlated with poorer OS of breast cancer patients compared to those with lower SMAD3 expression (Fig. 8G). Furthermore, KLLN mRNA levels were significantly reduced in the TNBC specimens compared to the normal breast tissues. Higher expression of KLLN correlated with better survival rates of TNBC patients (Fig. 8H, I). Besides, KLLN expression showed negative correlation with miR-3940-3p expression in the breast cancer samples (Fig. 8J).

Taken together, these results suggested that SMAD3 inhibited transcription of SEAS1, which acted as a sponge for miR-3940-3p, thereby regulating the expression of the tumor suppressor gene KLLN (Fig. 8K).

## Discussion

Accumulating evidence revealed that lncRNAs play important functions in the regulation of genes that control tumor proliferation, apoptosis, and migration, and expanded our understanding of the biological behavior of disease, particularly in cancers including breast cancer. Several studies have demonstrated the functions of multiple anti-sense lncRNAs in breast cancer. Anti-sense lncRNAs play a significant role in the epigenetic regulation of gene transcription by fine-tuning histone modifications or modulating the bioavailability of their target miRNAs. For example, lncRNA MAGI2-AS3 expression correlated negatively with worse prognosis in breast cancer because MAGI2-AS3 regulated DNA methylation of MAGI2 by blocking the Wnt/b-catenin pathway [23]. LncRNA PHACTR2-AS1 modulated breast cancer growth and metastasis by maintaining the H3K9 methylation-marked silent state of the ribosomal DNA genes [24]. Our findings demonstrated that the novel tumor-suppressive antisense lncRNA SEAS1 regulated breast tumor growth and aggressiveness. Therefore, SEAS1 is a promising therapeutic target for breast cancer.

SEAS1 is an important suppressor of breast cancer proliferation and is expressed at relatively low levels in almost all the major tissues, thereby indicating its essential role in several tissues. Zhong et al. reported that SEAS1 decreased HCC cell proliferation and progression by suppressing miR-718 through upregulation of PTEN [25]. SEAS1 also acted as a tumor suppressor gene in ESCC [26], gastric cancer [27], and human mesenchymal stem cells [28]. However, very few reports are available on the expression of SEAS1 in TNBC. We found that SEAS1 levels were significantly decreased in breast cancer samples through the examination of TCGA databases and this trend was further confirmed in TNBC tissues. The low expression of SEAS1 correlated with poor prognosis in the TNBC patients. Functional studies showed that SEAS1 inhibited proliferation, migration, invasiveness, and accelerated apoptosis of TNBC cells, both in vitro and in vivo, thereby suggesting its tumor-suppressor role in breast cancer.

We identified miR-3940-3p binding sites in the SEAS1 sequence based on bioinformatics analyses and confirmed their interaction using luciferase reporter and RIP assays. Furthermore, the expression of SEAS1 showed negative correlation with the expression of miR-3940-3p in the TNBC tissues and cell lines. We also demonstrated the existence of a feedback inhibitory loop between SEAS1 and miR-3940-3p in the breast cancer cells. The miRNAs are a class of small endogenous non-coding RNA molecules of about 20-25 nucleotides in length that post-transcriptionally regulate gene expression through targeted mRNA excision or translational repression by pairing with the 3′ untranslated region (3′UTR) of their target mRNAs [29]. The miRNAs play a critical role in several biological processes including cell proliferation, apoptosis, and differentiation [30, 31]. In the present study, we demonstrated that miR-3940-3p was regulated by SEAS1. MiR-3940-3p inhibitor could reverse the effect of SEAS1 silence on cell proliferation, apoptosis, migration, and invasion. This suggested that SEAS1 modulated TNBC progression by regulating miR-3940-3p levels.

We then showed that TNBC progression was regulated by the SEAS1/miR-3940-3p axis via KLLN. Hu et al. showed that miR-224 promoted ovarian cancer cell proliferation by targeting KLLN [32]. Wang et al. [33] reported that KLLN induced cell cycle arrest and apoptosis in breast cancer cells by directly promoting the expression of TP53 and TP73. Wang et al. [34] demonstrated that KLLN promoted prostate cancer cell apoptosis by upregulating TP53 and TP73. In NSCLC, KLLN functioned as a tumor suppressor gene by regulating the p53 signaling pathway and was targeted by miR-149-3p and miR-4270 [35]. We demonstrated that KLLN levels were significantly reduced in the TNBC tissues. KLLN silencing promoted migration, invasion, survival, and proliferation of the TNBC cells. Hence, this study demonstrated that KLLN was a tumor suppressor in TNBC cells. We also demonstrated that KLLN was post-transcriptionally regulated by SEAS1 in TNBC cells and tissues.

SMAD3 is a member of the SMAD family and the TGF-β superfamily and mediates signal transduction pathways regulating cell proliferation, apoptosis, immune surveillance, and cancer metastasis [36]. Phosphorylated SMAD3 forms oligomeric complexes with SMAD4 through the TGF-β receptor and/or SMAD2 [37]. The oligomeric complexes with SMAD3 translocate to the nucleus, bind to the promoters of target genes such as IL-6 and CXCL2, and promote metastasis through recruitment of the MDSCs [38–40]. We demonstrated through RNA pull down experiments that SMAD3 was bound to SEAS1 as an RBP. The online GeneCards website reported that SMAD3 is widely distributed in several cell types. The online PROMO database showed that SMAD3 was a transcription factor binding to the SEAS1 promoter. Moreover, Yu et al. [41] reported that SMAD3 acted as an oncogene in TNBC through the KAT6A acetylation-dependent regulatory mechanism. In conclusion, our data demonstrated a relationship between SMAD3, SEAS1, and miR-3940-3p in TNBC cells.

In summary, this study demonstrated that lncRNA SEAS1 was a novel tumor suppressor in breast cancer. Reduced expression of lncRNA SEAS1 correlated with enhanced tumor metastasis and poor prognosis in breast cancer patients. LncRNA SEAS1 promoted KLLN expression by sponging miR-3940-3p. Furthermore, SMAD3 inhibited SEAS1 transcription by binding directly to its promoter and suppressing its transcription. Our results demonstrated that SEAS1 was a potential therapeutic target and prognostic predictor in breast cancer.

## Materials and methods

### Patients and specimens

Human breast cancer tissues and adjacent normal (peritumor) tissues were obtained at the surgery in Union Hospital of Tongji Medical College from October 2020 to May 2021. All participants provided written informed consent, and the research was approved by the Ethical Committee on Scientific Research of Union Hospital of Tongji Medical College. Fresh tumor tissues were validated by pathological diagnosis, frozen in liquid nitrogen, and stored at −80 °C.

### Cell culture and reagents

Human triple-negative breast cancer cell line MDA-MB-231 and BT-549 were purchased from the American Type Culture Collection (ATCC, USA). Cells were cultivated in RPMI 1640 medium (GIBCO, USA), which was supplemented with 10% fetal bovine serum (FBS) (GIBCO, USA) at 37 °C humidified condition containing 5% CO_2_.

### RNA sequencing analysis

Data covered the information of triple-negative breast cancer gene expression were downloaded from TCGA dataset. R software using the DEGseq package performed the data analysis. The threshold set for significant differences was log2|fold change| ≥ 1 and *P*-value <0.05.

### RNA extraction and quantitative real-time PCR analysis

Total RNA was extracted according to the manufacturer’s protocol using RNAiso Plus reagent (TaKaRa, Japan). mRNAs were reverse transcribed to Complementary DNA (cDNAs) using a PrimeScript RT Master Mix Kit (TaKaRa, Japan). The mRNA levels were detected by quantitative real-time PCR with an SYBR Premix Ex TaqII Kit (TaKaRa, Japan) in triplicate using synthesized primers (Tsingke, China). Relative RNA abundances (GAPDH as an internal control for mRNA, U6 as an internal control for miRNA) were calculated by the standard 2^−ΔΔ^Ct method. The sequences of siRNAs and mimics, as well as primers used in the study, are listed in Supplementary Tables 1 and 2.

### Subcellular fractionation

Nuclear and cytoplasmic separation was carried out with the PARIS Kit (Life Technologies, USA) according to the manufacturer’s instructions.

### RNA fluorescence in situ hybridization (RNA-FISH)

The FISH assay was performed in MDA-MB-231 cells. Antisense or sense probe for SEAS1 junction sequence was synthesized. Hybridization was undertaken using a Fluorescent In Situ Hybridization Kit (RiboBio, Guangzhou, China). The probe sequences for FISH were showed in Supplementary Table 3.

MDA-MB-231 cells were seeded in glass chamber slides. 24 h after transfection, cells were fixed in 4% paraformaldehyde (Thermo Scientific, Rockford, IL, USA) for 10 min, washed three times with PBS (5 min/wash, Sigma-Aldrich, USA), permeabilized with 0.5% Triton X-100/PBS permeation fluid at room temperature for 20 min, and again washed three times with PBS (5 min/wash). After permeabilization, the cells were incubated with specific probes at 37 °C overnight. The cell nucleus were stained with DAPI. The staining results were observed using a confocal fluorescence microscope.

### Plasmid construction and stable transfection

The full-length lncRNA SEAS1 cDNA was cloned into pcDNA3.1 (Invitrogen, USA). siRNAs and plasmids were transfected into breast cancer cells by applying Lipofectamine 3000 (Invitrogen) according to the manufacturer’s instructions. Quantitative real-time PCR was used to evaluate the transfection efficiency.

### CCK8 assay

We also carried out a CCK8 assay according to the manufacturer’s protocol to evaluate the proliferation efficiency of the SEAS1 (miR-3940-3p, KLLN) for triple-negative breast cancer cells. MDA-MB-549 and BT-549 were seeded into 96-well plates at a density of 1000 cells/well. RPMI-1640 (or L-15) containing 10% CCK8 solution (Dojindo, Japan) was added to each well and incubated for 1 hour in a 37 °C incubator. The spectral absorbance of each well at 450 nm was measured on a microplate reader (Thermo Fisher, USA) to assess the efficiency of cell proliferation.

### Colony formation assay

Two cell lines (1000 cells/well) after relevant transfection were seeded into 6-well plates and incubated at 37 °C using RPMI 1640 (L-15) medium with 10% FBS. 10–14 days later, cell colonies were washed with PBS, fixed with 4% paraformaldehyde (Thermo Scientific, Rockford, IL, USA) for 15 min, and stained with 0.2% crystal violet (Solarbio) for 20 min. The colonies were imaged and counted.

### EdU incorporation assay

EdU proliferation assays were carried out with an EdU incorporation assay kit (RiboBio, China) according to the manufacturer’s instructions. Transfected cells (2 × 10^4^ cells/well) were seeded into 96-well plates. After 24 h, 100 μl medium containing 50 mM EdU was added to each well and then incubated for 2 h at 37 °C. Cells were then fixed with 4% paraformaldehyde and stained with Hoechst and Apollo reaction cocktail.

### Flow cytometry analysis

Annexin V-fluorescein isothiocyanate (FITC)/propidium iodide (PI) Apoptosis Detection Kit (Beyotime) and Annexin V-APC (eBioscience) kit were used to determine cell apoptosis. We washed the cell pellet with 4 °C pre-cooled PBS 1 time and 1× binding buffer once (1500 rmp, 5 min centrifuged). Cell precipitation was resuspended by 200 µL 1 × binding buffer and added 4 µL Annexin V-FITC/PI [Annexin V-APC (eBioscience)] staining for 15 min at room temperature away from light. According to the amount of cells, added 100 µL 1 × binding buffer for flow cytometry analysis.

### Transwell assays

Transwell insert chambers (Scipu001412; Corning, Corning, NY, USA) pre-coated with (for cell invasion assay) or without (for cell migration assay) Matrigel (Corning Inc.) were utilized to evaluate the invasion and migration of breast cancer cells. In brief, about 2 × 10^4^ transfected cells suspended in 200 μL serum-free RPMI1640 (L-15 for MDA-MB-231) were added into the upper chamber and 500 μL culture medium with 20% FBS was added into the bottom chamber. After 24 h, migrated/invaded cells were fixed with 4% paraformaldehyde (Sangon), stained with crystal violet (Solarbio) and then observed with an inverted microscope. Photographs of 5 randomly selected fields were taken and migrated/invaded cell number per field = the total count of five high power fields/5.

### Western blot assay

Total protein was isolated with RIPA buffer (Beyotime), determined with a BCA protein assay kit (Beyotime). After SDS-PAGE electrophoresis and NC membrane (0.2 μm, New York, USA) transfer, NC membranes were blocked with 5% skim milk powder and incubated with specific antibodies at 4 °C overnight. The membranes were incubated with secondary antibodies. ECL detection system (Bio-Rad, USA) was used to detect the protein bands. Primary antibodies included Caspase3 (9662p; 1:1000; CST), caspase7 (9492p; 1:1000; CST), bax (5023t; 1:1000; CST), bcl2 (a19693; 1:1000; Abclon), parp (a19596; 1:1000; Abclon), KLLN (ab197892; 1:1000; Abcam), GAPDH (ab181602; 1:10,000; Abcam), anti-cyclin A2 (91500; 1:1000; CST).

### Tumor xenograft model

All animal experiments were carried out following NIH Guidelines for the Care and Use of Laboratory Animals and approved by the Animal Care Committee of Tongji Medical College. MDA-MB-231 cells (10^7^ cells) with or without lncRNA SEMA3B-AS1 overexpression were suspended in 200 μl PBS and subcutaneously injected into each flank of 4–6-week-old BALB/c nu female mice (three mice per group). The mice were sacrificed after 5 weeks, and the maximum (*L*) and minimum (*W*) length and weight of the tumors were measured. Tumor volume was calculated as ½*LW*^2^. Tumors were detected by hematoxylin and eosin (H&E) and immunohistochemical staining.

### Protein was extracted from tissue homogenate

After harvesting the patients’ tumor tissues as well as xenograft samples from mice, they were frozen immediately in liquid nitrogen and stored at −80 °C subsequently. The process of tissue homogenate was as follows: (1) Break a big piece of frozen tissue into small pieces and transfer them into a 2 mL microcentrifuge tube. (2) Add 5 vol of extraction buffer to the tube. (3) Homogenize the tissue with 10 to 15 strokes (3–4 s/stroke) using a mini-homogenizer and plastic pestle on ice. (4) Spin at 12,000 × *g* for 15 min at 4 °C. (5) Transfer the supernatant to a fresh tube. Try not to take any lipid from the surface layer or any precipitated particle from the bottom. (6) Spin again at 12,000 × *g* for 10 min at 4 °C. (7) Transfer supernatant to a fresh tube.

### Immunohistochemistry

Immunohistochemical staining and quantitative evaluation were performed with antibodies specific for Ki-67 (ab16667; 1:200; Abcam) and PCNA (ab29; 1:10,000; Abcam). The degree of positivity was measured according to the percentage of positive cancer cells.

### Dual-luciferase reporter assay

The fragments of SEAS1 and KLLN 3′ UTR containing the predicted binding sites of wild-type or mutant miR-3940-3p were inserted into the pmirGLO plasmid (Promega, Madison, WI, USA) to construct the luciferase reporter vectors SEAS1-wt, SEAS1-mut, KLLN-wt, and KLLN-mut, respectively. Then MDA-MB-231 cells were seeded into 24-well plates (10^5^ cells/well) and transfected with miR-NC or miR-3940-3p together with corresponding luciferase reporter vector.

The SEAS1 promoter region (−2000 bp to +100 bp sequence upstream of transcription initiation site) containing the assumed binding region or mutation region was constructed into the vector based on pGL3 (named pGL3-SEAS1) and transfected into TNBC cells. After 48 h, Dual-Luciferase Reporter Assay Kit (Promega) was used to measure the luciferase activity.

### RNA immunoprecipitation (RIP) assay

A Magna RIP RNA-Binding Protein Immunoprecipitation Kit (Millipore, USA) was used to determine the relationship between SEAS1 and miR-3940-3p. Antibodies used for the RIP assay included anti-AGO2 and control IgG (Millipore, USA), and the coprecipitated RNAs were used for cDNA synthesis and evaluated by qRT-PCR.

### Chromatin immunoprecipitation (ChIP)

ChIP assay was performed using EZ-Magna ChIP A/G (17-10086, Upstate, Millipore, MA, USA) kit according to the manufacturer’s instructions. Antibodies against samd3 were used for immunoprecipitation. Quantitative analysis of ChIP-derived DNA was performed by PCR reaction.

### GEPIA tool analysis and PROMO database

The GEPIA (http://gepia.cancer-pku.cn/index.html) was used to determine the association between SMAD3, SEAS1, and KLLN and the prognosis of breast cancer patients. The online PROMO database (http://alggen.lsi.upc.es/cgi-bin/promo_v3/promo/promoinit.cgi?dirDB=TF_8.3/) predicts the promoter bound TF.

### RNA pull-down and mass spectrometry

Double-stranded SEAS1 promoter was synthesized by PCR and labeled with biotin-14-dCTP under the manufacturer’s protocol (Invitrogen, Grand Island, NY, USA). Biotin-labeled DNA was diluted in 10 mM Tris-HCl, 10 mM MgCl_2_, 25 mM NaCl, and 10% glycerol and incubated with nuclear protein extract at 4 °C for 12 h. In general, the biotinylated DNA was pulled down by streptavidin-linked magnetic beads (Thermo Scientific) at room temperature for 2 h. The beads containing DNA and proteins were then washed four times with 1× binding and washing buffer. The proteins were precipitated and diluted in 100 μl of protein lysis buffer. One-shot mass spectrometry analyses were then performed to analyze the purified nuclear proteins. Liquid chromatography-mass spectrometry (LC-MS) (Novogene, Beijing, China) was used for mass spectrometry analyses.

### TUNEL staining

TUNEL assay was performed using an In Situ Cell Death Detection Kit (Roche, Mannheim, Germany) according to the provided protocol. Briefly, following less washing in Phosphate-buffered saline (PBS), the cells were fixed in 4% paraformaldehyde solution and treated with 0.1% Triton ×100 solution (Sigma, Germany) for 3 min. Then, the cells were primarily incubated in TUNEL solution (Roche, Germany) at 37 °C for an hour according to the manufacturer’s instructions.

### Statistical analysis

SPSS v.21.0 software (IBM, Armonk, NY, USA) was applied for statistical analyses. Mean ± SD was used to present experimental results. Student’s *t*-test or one-way ANOVA was used to detect the differences among groups and expression of LncRNA SEMA3B-AS1 and the clinical characteristics were analyzed by chi-square test. *p* values <0.05 were considered statistically significant.

### Reporting summary

Further information on research design is available in the Nature Research Reporting Summary linked to this article.

## Supplementary information

Supplementary Figure 1

Supplementary Figure 2

Supplementary Figure 3

Supplementary Figure 4

Supplementary Figure 5

Supplementary Figure 6

Supplementary Figure 7

Supplementary Figure 8

Supplementary Table 1

Supplementary Table 2

Supplementary Table 3

Supplementary Table 4

Supplementary Legend

Reporting Summary

Original Data File

## Data Availability

All data generated or analyzed during the present study are included in this published article or are available from the corresponding author on reasonable request.
