# Supplementary material for: LncRNA SEMA3B-AS1 inhibits breast cancer progression by targeting miR-3940/KLLN axis
Source: Cell Death Dis. 2022 Sep 19;13(9):800. doi: 10.1038/s41419-022-05189-7 (PMC9485163; doi:10.1038/s41419-022-05189-7)
Supplement: Supplementary file 9 — Supplementary Table 1 [file 41419_2022_5189_MOESM9_ESM.docx]

Supplementary Table 1 The sequences of siRNAs and mimics.

| **SiRNA Targets** | **Sequences** |
| --- | --- |
| SEMA3B-AS1-siRNA#1 sense | 5′-GCAAAGGGAATCACTGAGT-3 |
| SEMA3B-AS1-siRNA#2 sense | 5′-GAAGTCCACCTGAGTGAAT-3′ |
| SEMA3B-AS1-siRNA#3 sense | 5′-TGAGGGAGTTTTACAGACA-3′ |
| KLLN-siRNA#1 sense | 5′- GTGAACACATAGCCGTTGAATTT-3′ |
| KLLN-siRNA#2 sense | 5′- ACCTATCTAAATGAACTGAAAGA-3′ |
| KLLN-siRNA#3 sense | 5′- TGCTCTACATCGACCTATTCTGC-3′ |
| Smad3-siRNA sence | 5’-AGUCAGUUGCAUUCAUUAAAUCAAC-3’ |
| FUS-siRNA#1 sense | 5′-AGGATAATTCAGACAACAACACC-3′ |
| FUS-siRNA#2 sense | 5′-GGGTGAGAATGTTACAATTGAGT-3′ |
| FUS-siRNA#3 sense | 5′-GTGAGAATGTTACAATTGAGTCT-3′ |
| FUBP1-siRNA#1 sense | 5′-ATCACTGAATTCAAATGACTATG-3′ |
| FUBP1-siRNA#2 sense | 5′-ATGGTTATGGGGGACAAAAAAGA-3′ |
| FUBP1-siRNA#3 sense | 5′-GGGACAAAAAAGACCTTTAGAAG-3′ |
| SFPQ-siRNA#1 sense | 5′-AGGGGTTTAAAGCCAATTTGTCT-3 |
| SFPQ -siRNA#2 sense | 5′-GGGTTTAAAGCCAATTTGTCTCT-3′ |
| SFPQ-siRNA#3 sense | 5′-GAGGATGAATTCAAAAGACTATT-3′ |
| NC-siRNA sense | 5′-UUCUCCGAACGUGUCACGUUU-3′ |
| NC mimics | 5′-CUCGGGCCAUCCCAGCCCACUU-3 |
| miR-3940-3p mimics | 5′-CAGCCCGGAUCCCAGCCCACUU-3′ |
| NC inhibitor | 5′-AAGUGGGCUGGGAUGGCCCGAG-3′ |
| miR-3940-3p inhibitor | 5′-AAGUGGGCUGGGAUCCGGGCUG-3′ |
