# Supplementary material for: LncRNA SEMA3B-AS1 inhibits breast cancer progression by targeting miR-3940/KLLN axis
Source: Cell Death Dis. 2022 Sep 19;13(9):800. doi: 10.1038/s41419-022-05189-7 (PMC9485163; doi:10.1038/s41419-022-05189-7)
Supplement: Supplementary file 10 — Supplementary Table 2 [file 41419_2022_5189_MOESM10_ESM.docx]

Supplementary Table 2 The sequences of PCR primers

| Primers | Sequences |
| --- | --- |
| SEMA3B-AS1 | Forward: 5′-GGAGGTGGGAGGAACAAC-3′ |
|  | Reverse: 5′-ACTTCAGGGCTCCACTCT-3′ |
| KLLN | Forward: 5′-ATGGATCGCCCGGGGCCAG-3′ |
|  | Reverse: 5′-TCAGTCCTTTGGCTTGCTCTTA-3′ |
| BIRC5 | Forward: 5′-AGGACCACCGCATCTCTACAT-3′ |
|  | Reverse: 5′-AAGTCTGGCTCGTTCTCAGTG-3′ |
| ALDH4A1 | Forward: 5′-GACGTGCAGTACCAAGTGTC-3′ |
|  | Reverse: 5′-GATCACGGTCTTACCCTGTCC-3′ |
| NDUFA11 | Forward: 5′-GCCGAAGGTTTTTCGTCAGTA-3′ |
|  | Reverse: 5′-GGAGGATTGAGTGTGACTCTGT-3′ |
| CLCN7 | Forward: 5′-CCCACACAACGAGAAGCTCC-3′ |
|  | Reverse: 5′-ACTTGTCGATATTGCCCTTGATG-3′ |
| GPRIN2 | Forward: 5′- CAGAGGAGCCATTCAGACCTG-3′ |
|  | Reverse: 5′- CACTGACAACTGACTCGCC-3′ |
| ARHGEF7 | Forward: 5′- TGCTTTCAACGTACCTACGGC -3′ |
|  | Reverse: 5′- GGCAACTTGGTGCATTCTTCTAA -3′ |
| METTL2A | Forward: 5′- GCAGTCCTCGCCGATAAGAG -3′ |
|  | Reverse: 5′-CTTCCGACCACTCCACATTGT-3′ |
| CEP170B | Forward: 5′- CGGACCAGAAGTACGTCACG-3′ |
|  | Reverse: 5′- CACACGCTCCAGCACATACA-3′ |
| PITPNM3 | Forward: 5′- TCGCTTGTCTCTCACCTGAAC-3′ |
|  | Reverse: 5′- CAGGAACTCTCTGTAGACCTGG-3′ |
| EXOSC6 | Forward: 5′- ACGCGGGAGTGGAGATGTA-3′ |
|  | Reverse: 5′- CGGTCCAACTCTCAGTCTGC-3′ |
| miR-3940-3p | Forward: 5′-CTCAAGGACCACCGCATC-3′ |
|  | Reverse: 5′- CAGTGCGTGTCGTGGAGT -3′ |
| miR-365a-3p | Forward: 5′-gcgTAATGCCCCTAAAAATC-3′ |
|  | Reverse: 5′- CAGTGCGTGTCGTGGAGT -3′ |
| miR-513c-5p | Forward: 5′-GGGTTCTCAAGGAGGTGTCG -3′ |
|  | Reverse: 5′- CAGTGCGTGTCGTGGAGT -3′ |
| miR-760 | Forward: 5′-GCGGCTCTGGGTCTGT -3′ |
|  | Reverse: 5′- CAGTGCGTGTCGTGGAGT -3′ |
| GAPDH | Forward: 5’-CCCCGGTTTCTATAAATTGAGC-3’ |
|  | Reverse: 5’-CACCTTCCCCATGGTGTCT-3’ |
| U6 | Forward: 5’-TTATGGGTCCTAGCCTGAC-3’ |
|  | Reverse: 5’-CACTATTGCGGGTCTGC-3’ |
| Cyclin A | Forward: 5’-CGCTGGCGGTACTGAAGTC-3’ |
|  | Reverse: 5’-GAGGAACGGTGACATGCTCAT-3’ |
