# Supplementary material for: LncRNA SEMA3B-AS1 inhibits breast cancer progression by targeting miR-3940/KLLN axis
Source: Cell Death Dis. 2022 Sep 19;13(9):800. doi: 10.1038/s41419-022-05189-7 (PMC9485163; doi:10.1038/s41419-022-05189-7)
Supplement: Supplementary file 11 — Supplementary Table 3 [file 41419_2022_5189_MOESM11_ESM.docx]

**Supplementary Table 3 The probe sequences for FISH**

LncRNA-SEMA3B-AS1 sence：

AACCCCAGCTCCAGGGGCTCAGCAGGCAAAGGGAATCACTGAGTGGGGGCACCACCCGTGGACTCCAATATCTCAACCTCTCCCTCCACAGGTTGGAGGTGGGAGGAACAACCCCCACCAAACCCAGAGCCGAAAACTGAGGGAGTTTTACAGACAGGACGGAGCTCCTGCACCTCGGAGCCTCAGTTGGGAATGACCTGGGGTCTTGTCCTGAAGCTGAGTCTGGTGAACGTGCCCCATTTGTAACATGAGGGGTACTTCTCTGGAGGGACTGTATGTTGACAGTGGCAGAGTGGAGCCCTGAAGTCCACCTGAGTGAATATACCAGGGCTTGAGAAAAAAAAAAAAAAAAAAAAA

LncRNA-SEMA3B-AS1 anti-sence：

TTTTTTTTTTTTTTTTTTTTTCTCAAGCCCTGGTATATTCACTCAGGTGGACTTCAGGGCTCCACTCTGCCACTGTCAACATACAGTCCCTCCAGAGAAGTACCCCTCATGTTACAAATGGGGCACGTTCACCAGACTCAGCTTCAGGACAAGACCCCAGGTCATTCCCAACTGAGGCTCCGAGGTGCAGGAGCTCCGTCCTGTCTGTAAAACTCCCTCAGTTTTCGGCTCTGGGTTTGGTGGGGGTTGTTCCTCCCACCTCCAACCTGTGGAGGGAGAGGTTGAGATATTGGAGTCCACGGGTGGTGCCCCCACTCAGTGATTCCCTTTGCCTGCTGAGCCCCTGGAGCTGGGGTT
