# Supplementary material for: LncRNA SEMA3B-AS1 inhibits breast cancer progression by targeting miR-3940/KLLN axis
Source: Cell Death Dis. 2022 Sep 19;13(9):800. doi: 10.1038/s41419-022-05189-7 (PMC9485163; doi:10.1038/s41419-022-05189-7)
Supplement: Supplementary file 12 — Supplementary Table 4 [file 41419_2022_5189_MOESM12_ESM.docx]

Supplementary Table 4 Clinicopathological correlations of lncRNA SEMA3B-AS1 expression in triple-negative breast cancer.

|  | **LncRNA SEMA3B-AS1 expression** | | | ***p*-value** |
| --- | --- | --- | --- | --- |
|  | **Low** | **High** | **total** |  |
| Age(y) |  |  |  |  |
| ≤ 60 | 22 | 27 | 49 | 0.381 |
| > 60 | 26 | 25 | 51 |  |
| Tumor size |  |  |  |  |
| ≤ 2 | 18 | 21 | 39 | 0.872 |
| > 2 and ≤ 5 | 24 | 24 | 48 |  |
| > 5 | 6 | 7 | 13 |  |
| Tumor differentiation |  |  |  |  |
| Well | 11 | 14 | 25 | 0.632 |
| Moderate | 22 | 25 | 47 |  |
| Poor | 15 | 13 | 28 |  |
| TNM stage |  |  |  |  |
| I~II | 33 | 29 | 62 | 0.082 |
| III~IV | 15 | 23 | 38 |  |
| Lymphatic metastasis |  |  |  |  |
| Positive | 29 | 12 | 41 | 0.031* |
| Negative | 19 | 40 | 59 |  |
| Distant metastasis |  |  |  |  |
| Positive | 14 | 8 | 22 | 0.001** |
| Negative | 34 | 44 | 78 |  |
