# Supplementary material for: LncRNA SEMA3B-AS1 inhibits breast cancer progression by targeting miR-3940/KLLN axis
Source: Cell Death Dis. 2022 Sep 19;13(9):800. doi: 10.1038/s41419-022-05189-7 (PMC9485163; doi:10.1038/s41419-022-05189-7)
Supplement: Supplementary file 13 — Supplementary Legend [file 41419_2022_5189_MOESM13_ESM.docx]

**Supplementary Figure Legend**

**Supplementary Figure 1.** GEPIA database analysis shows differential expression of seven lncRNAs including SEAS1 in the breast cancer tissues and normal breast tissues. Kaplan-Meier survival curves show the survival rates of breast cancer patients with high and low expression of these seven lncRNAs to determine their association with prognosis of TNBC patients.

**Supplementary Figure 2.** The sequence, secondary structure, and protein-coding capacity of SEAS1. (**A)** Schematic diagram shows the genomic locus of lncRNA SEAS1 in the human genome (Chromosome 3). The pink rectangles represent predicted exons in the SEAS1 sequence. (**B)** The nucleotide sequence of lncRNA SEAS1. (**C)** The secondary structure of lncRNA SEAS1 from the AnnoLnc database (http://annolnc.cbi.pku.edu.cn/). (**D)** Putative ORFs in the lncRNA SEAS1 sequence as predicted by the ORF Finder. (**E)** The predicted amino acid sequences of putative proteins derived from the lncRNA SEAS1 sequence. (**F)** Protein coding potential of the lncRNA SEAS1 sequence based on 5 different metrics.

**Supplementary Figure 3.** Characterization of SEAS1 and its regulation in breast cancer cell lines. (**A)** RT-qPCR analysis shows the levels of SEAS1 in MDA-MB-231 and BT-549 cells transfected with three different siRNAs against human SEAS1 (siSEAS1 ). (**B)** RT-qPCR analysis shows the levels of SEAS1 in MDA-MB-231 and BT-549 cells transfected with the lentiviral vector control and lentiviral vector containing the SEAS1 sequence. (C-D) RT-qPCR shows the levels of pri-miR-3940-3p (C) and pre-miR-3940-3p (D) in the control and SEAS1 -silenced MDA-MB-231 and BT-549 cells. (**E)** RIP assay results show the levels of SEAS1 and miR-3940-3p pulled down from the b xenograft tumor lysates the anti-Ago2 antibody and IgG control. (**F)** Schematic diagram shows the predicted wild-type and mutated binding sites for SMAD2 in the SEAS1 promoter sequence. (**G)** Luciferase reporter assay results show the relative luciferase activity in MDA-MB-231 and BT-549 cells co-transfected with the pGL3 luciferase reporter vector containing wild-type (SEAS1 -WT) or mutated (SEAS1 -MUT) SEAS1 promoter and SMAD3 expression vector or negative control (NC). The firefly luciferase activity was estimated at 48 h after transfection and normalized with the Renilla luciferase activity. (**H)** Luciferase reporter assay results show the relative luciferase activity in MDA-MB-231 and BT-549cells co-transfected with the pGL3 luciferase reporter vector containing wild-type (SEAS1 -WT) or mutated (SEAS1 -MUT) SEAS1 promoter and siSMAD3 or siNC. (**I)** RT-qPCR analysis shows the expression levels of SEAS1 in the case and control groups. *P< 0.05, **P< 0.01, and ***P< 0.001. Representative data from at least 2–3 experiments with comparable results are shown.

**Supplementary Figure 4.** LncRNA SEAS1 functions as a miR-3940-3p sponge in the BT-549 cells. (**A-C)** CCK8, colony formation, and EdU assay results show the proliferation rates of miR-3940-3p overexpressing or silenced BT-549 cells and the corresponding controls. (**D)** Flow cytometry analysis shows the apoptotic rates of miR-3940-3p overexpressing or silenced BT-549 cells and the corresponding controls. (**E)** Transwell assay results show the migration and invasion rates of miR-3940-3p overexpressing or silenced BT-549 cells and the corresponding controls. The data represent mean ± S.D of three independent experiments. *P< 0.05, **P< 0.01, and ***P< 0.001. Representative data from at least 2–3 experiments with comparable results are shown.

**Supplementary Figure 5.** SEAS1 silencing reverses the effects of miR‑3940-3p in the BT-549 cells. (**A-C)** CCK8, colony formation, and EdU assays demonstrate the proliferation rates of BT-549 cells co-transfected with siSEAS1 and miR-3940-3p inhibitor and the corresponding controls. (**D)** FACS analysis shows the apoptotic rates of BT-549 cells co-transfected with siSEAS1 and miR-3940-3p inhibitor and the corresponding controls. (**E)** Transwell assay results show the migration and invasion rates of BT-549 cells co-transfected with siSEAS1 and miR-3940-3p inhibitor and the corresponding controls. (**F)** Western blot analysis shows the expression levels of EMT-related proteins, namely, N-cadherin, Vimentin, Snail, and E-cadherin, in BT-549 cells co-transfected with siSEAS1 and miR-3940-3p inhibitor and the corresponding controls. *P< 0.05, **P< 0.01, and ***P< 0.001. Representative data from at least 2–3 experiments with comparable results are shown.

**Supplementary Figure 6.** KLLN silencing rescues the suppressive effects of the miR‑3940-3p inhibitor in the BT-549 cells. (**A-C)** CCK-8, colony formation, and EdU assay results show the proliferation rates of BT-549 cells co-transfected with the miR-3940-3p inhibitor and siKLLN and the corresponding controls. (**D)** Transwell assay results show the migration and invasion rates of BT-549 cells co-transfected with the miR-3940-3p inhibitor and siKLLN and the corresponding controls. (**E)** Representative western blot shows the expression levels of EMT-related proteins, namely, N-cadherin, Vimentin, Snail, and E-cadherin in the BT-549 cells co-transfected with the miR-3940-3p inhibitor and siKLLN and the corresponding controls. *P< 0.05, **P< 0.01, and ***P< 0.001. Representative data from at least 2–3 experiments with comparable results are shown.

**Supplementary Figure 7. (A)** RT-qPCR analysis shows the expression levels of SEAS1, miR-3940-3p, and KLLN mRNA in SMAD3-silenced and SMAD3-overexpressing TNBC cells and the corresponding controls. (**B)** Western blot analysis shows the levels of KLLN protein in SMAD3-silenced and SMAD3-overexpressing MDA-MB-231 and BT-549 cells. (C-D) CCK-8 assay shows the proliferation rates of SMAD3-silenced and SMAD3-silenced plus SEMA3B-AS1-silenced MDA-MB-231 and BT-549 cells. (**E-F**) Transwell assay results show the migration and invasion rates of SMAD3-silenced and SMAD3-silenced plus SEMA3B-AS1-silenced MDA-MB-231 and BT-549 cells. *P< 0.05, **P< 0.01, and ***P< 0.001. Representative data from at least 2–3 experiments with comparable results are shown.

**Supplementary Figure 8.** SMAD3 modulates TNBC progression by regulating the SEAS1 /miR-3940-3p/KLLN axis. (**A-C**) CCK8, colony formation, and EdU assay results demonstrate proliferation efficiency of TNBC cells co-transfected with siSMAD3 and miR-3940-3p mimics. (**D)** Flow cytometry analysis shows the apoptotic rates of TNBC cells co-transfected with siSMAD3 and miR-3940-3p mimics and the corresponding controls. (**E)** Transwell assay shows the migration rates of breast cancer cells co-transfected with siSMAD3 and miR-3940-3p mimics and the corresponding controls. *P< 0.05, **P< 0.01, and ***P< 0.001. Representative data from at least 2–3 experiments with comparable results are shown.

**Supplementary Table Legend**

**Supplementary Table 1.** The sequences of siRNAs and mimics.

**Supplementary Table 2.** The sequences of PCR primers.

**Supplementary Table 3.** The probe sequences for FISH.

**Supplementary Table 4.** Clinicopathological correlations of lncRNA SEMA3B-AS1 expression in triple-negative breast cancer.
