# Supplementary material for: LncRNA SEMA3B-AS1 inhibits breast cancer progression by targeting miR-3940/KLLN axis
Source: Cell Death Dis. 2022 Sep 19;13(9):800. doi: 10.1038/s41419-022-05189-7 (PMC9485163; doi:10.1038/s41419-022-05189-7)

**Original full length western blots**

**Figure 3F**

GAPDH

Snail

Vimentin

E-cadherin

N-cadherin

**
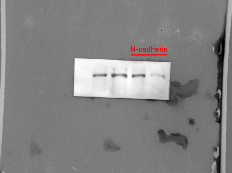

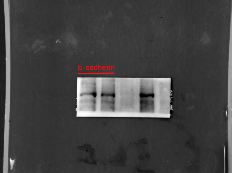

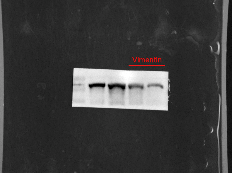

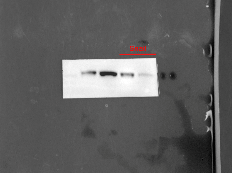

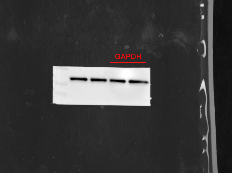
**


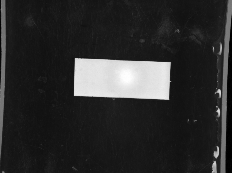

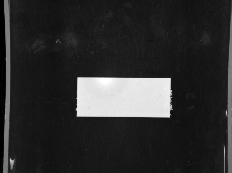

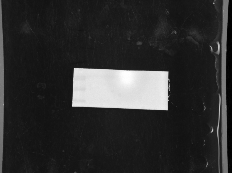

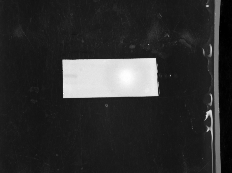

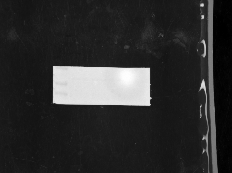


GAPDH

Snail

Vimentin

E-cadherin

N-cadherin

**
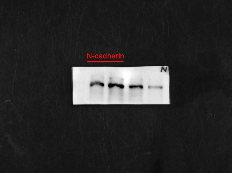

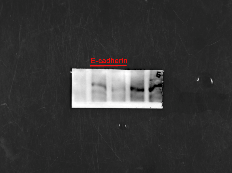

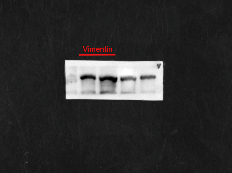

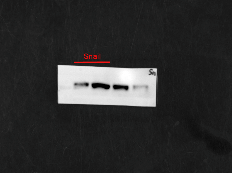

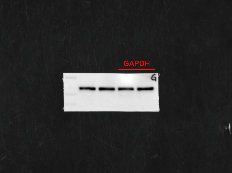
**

**
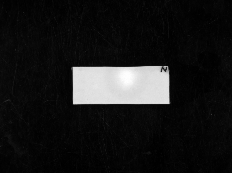

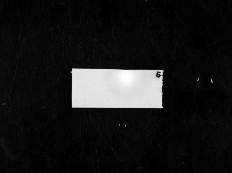

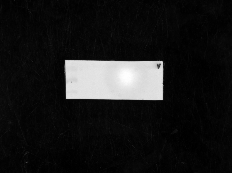

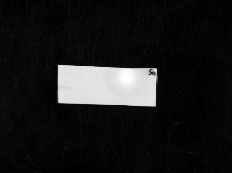

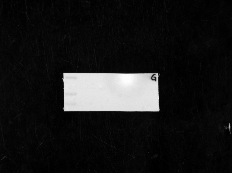
**

**Figure 5H**

**
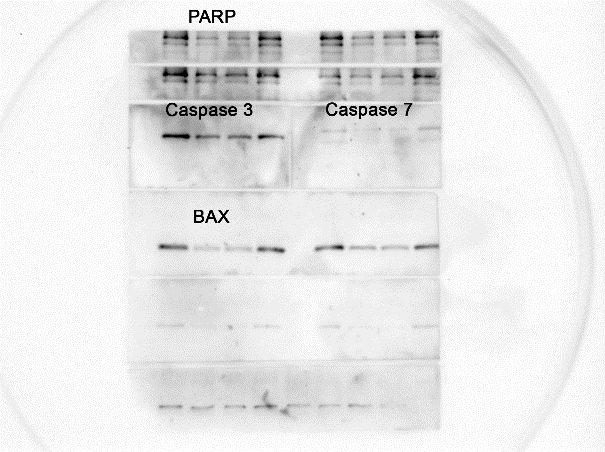

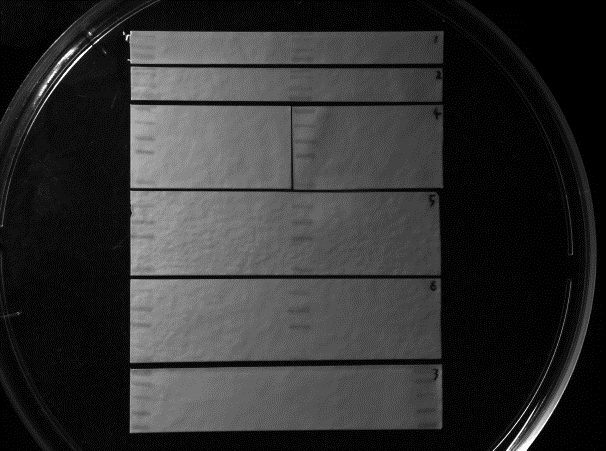
**

**
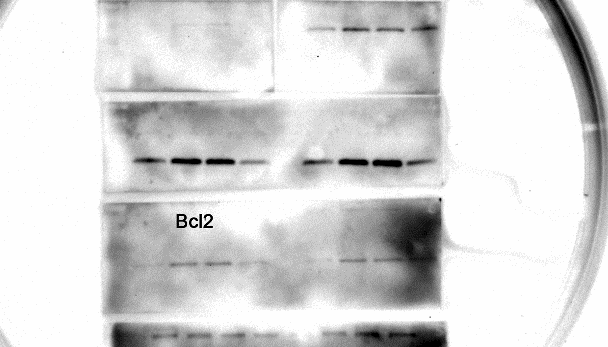

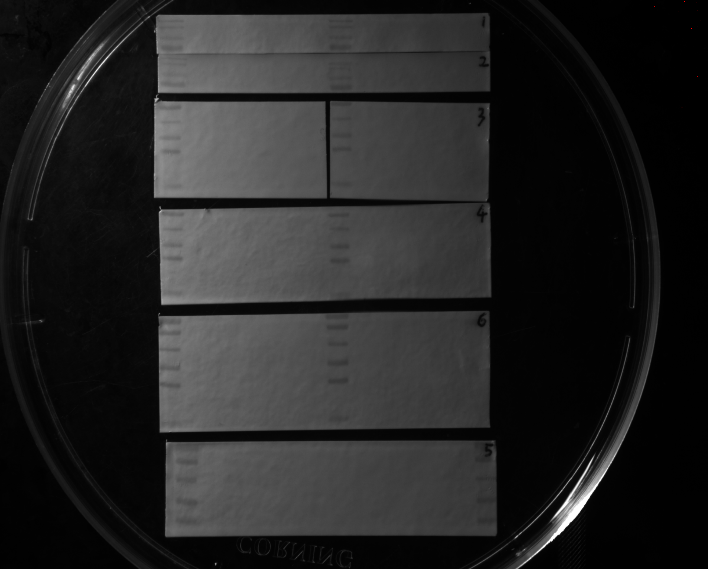
**

**
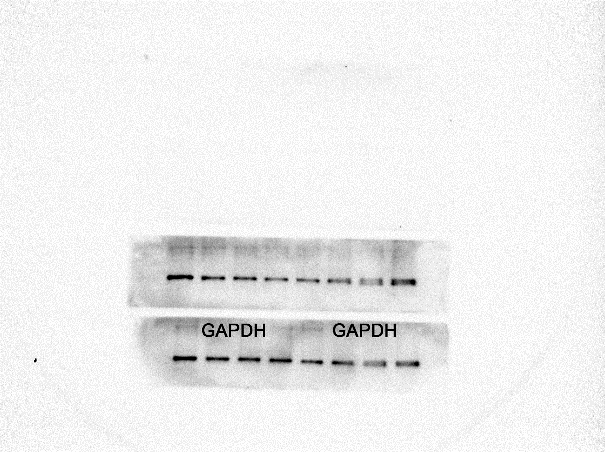

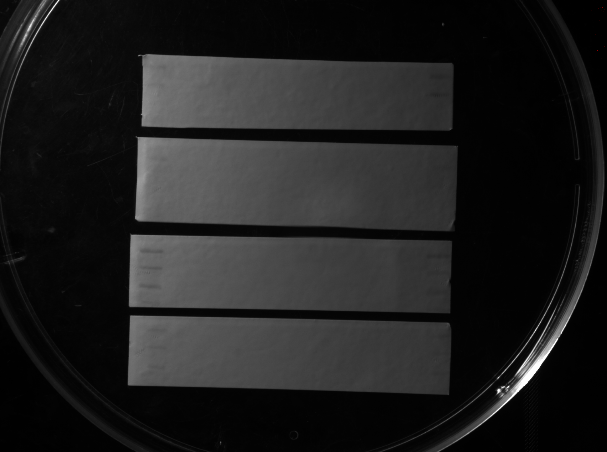
**

**Figure 5I**

GAPDH

Snail

Vimentin

E-cadherin

N-cadherin

**
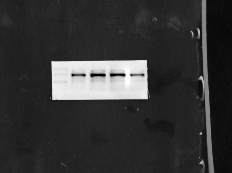

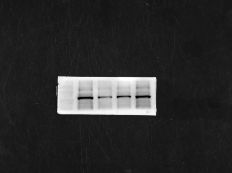

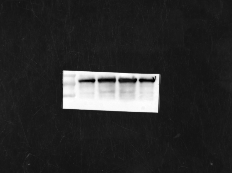

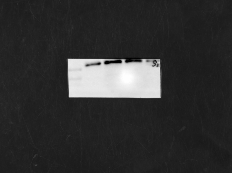

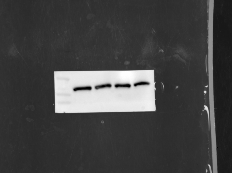
**

**
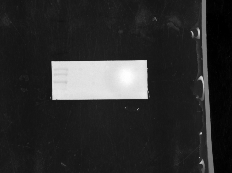

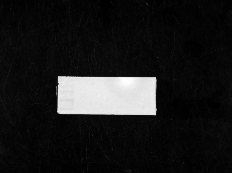

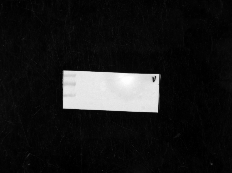

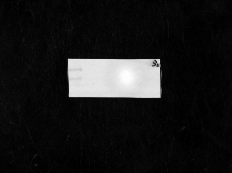

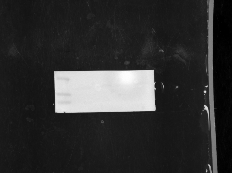
**

**Figure 6F**

**
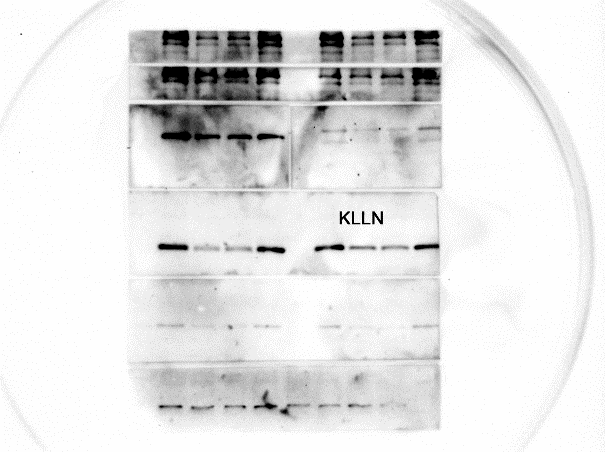

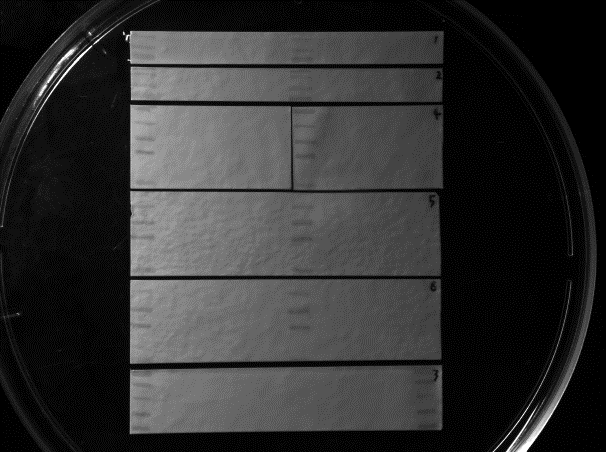
**

**
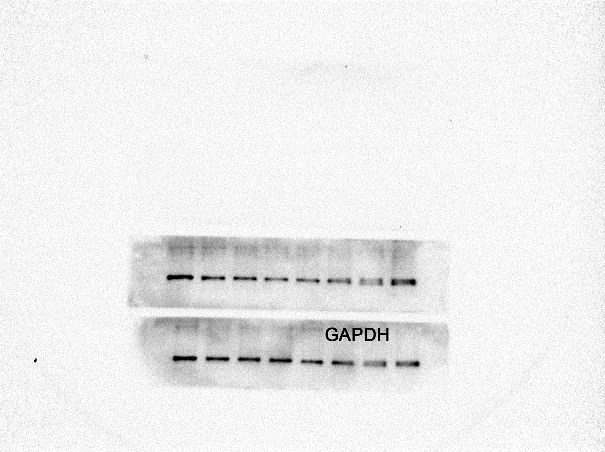

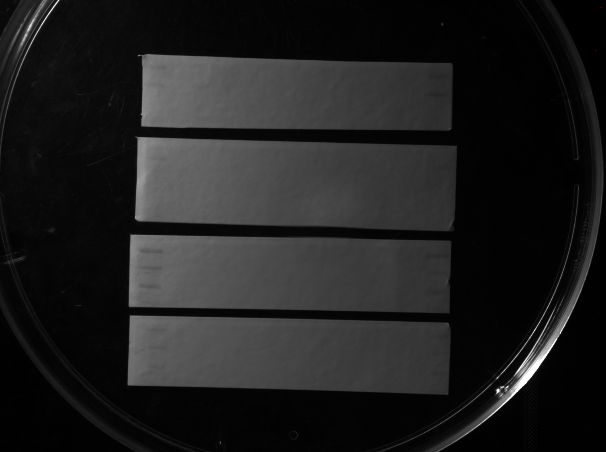
**

**Figure 6L**

**
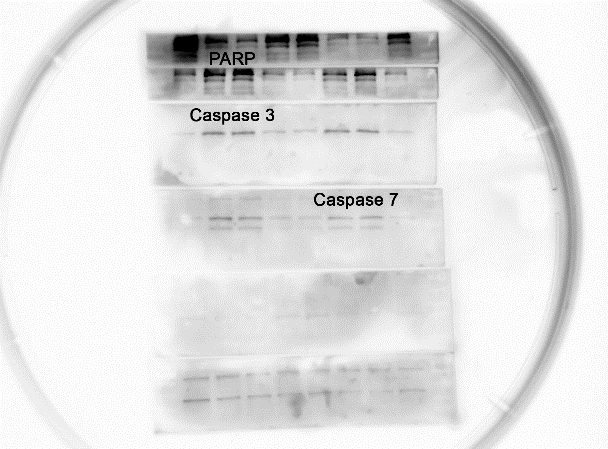

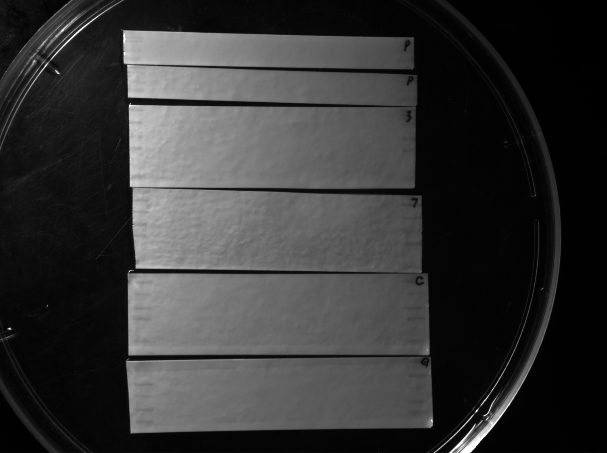
**

**
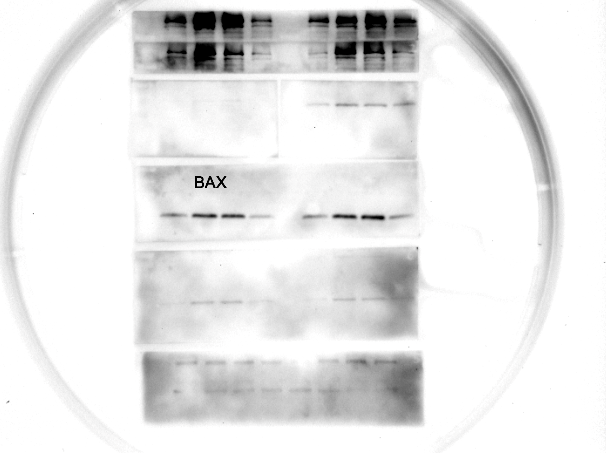

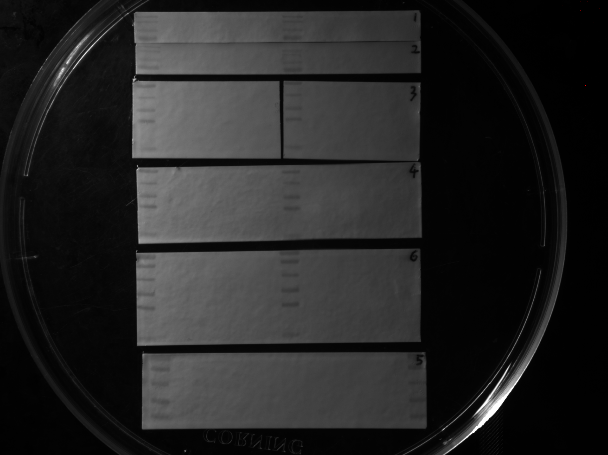
**

**
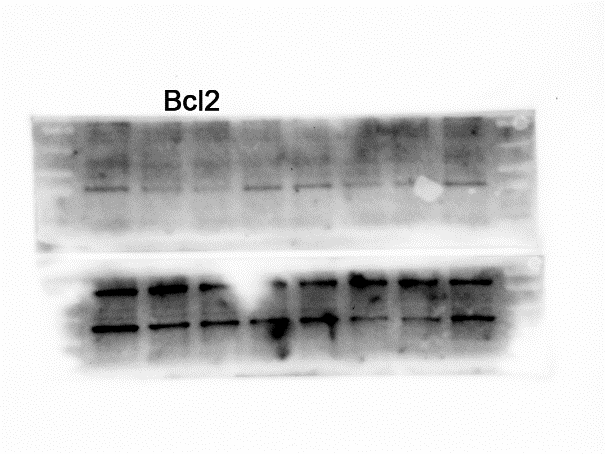

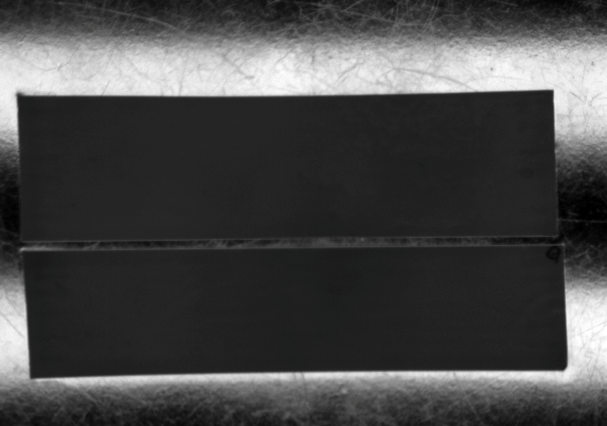
**

**
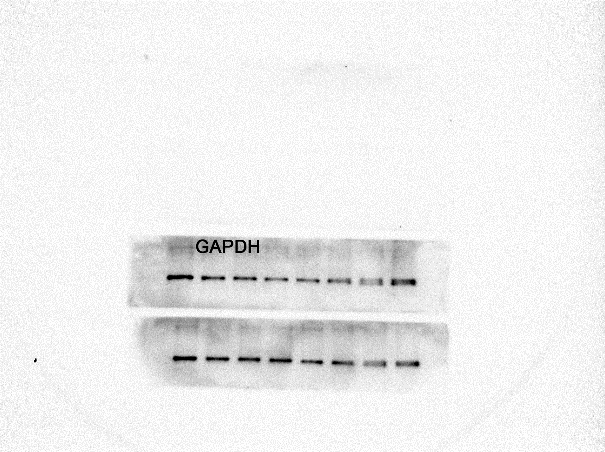

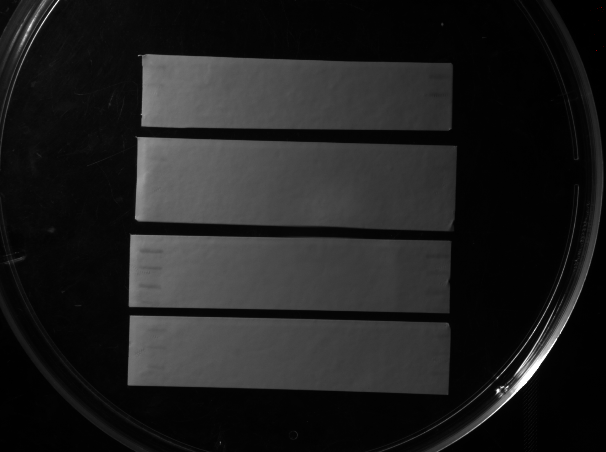
**

Figure 6M


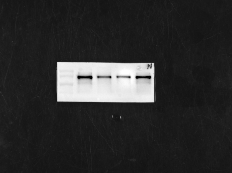

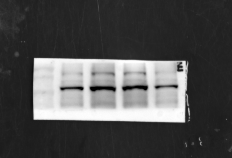

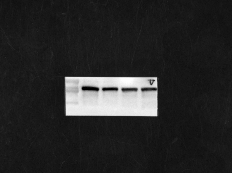

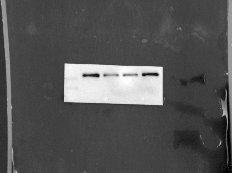

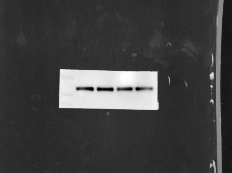


E-cadherin

Vimentin

Snail

GAPDH

N-cadherin


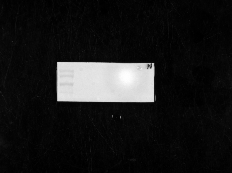

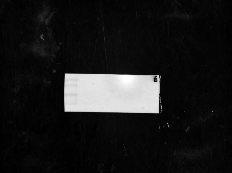

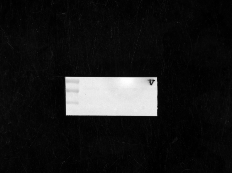

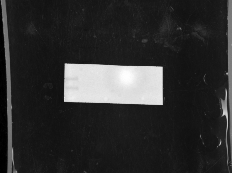

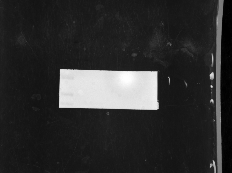


E-cadherin

Vimentin

Snail

GAPDH

Figure S5F

N-cadherin


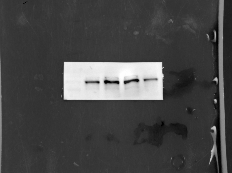

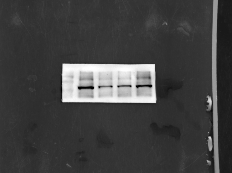

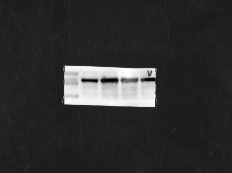

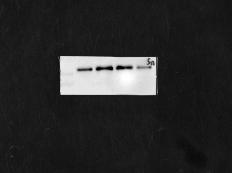

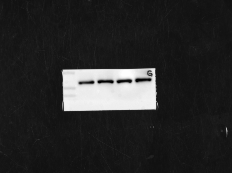


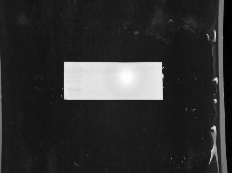

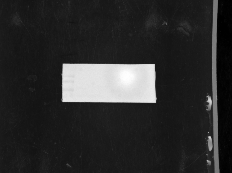

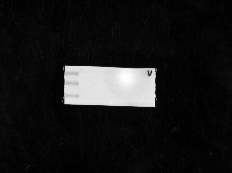

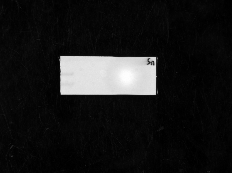

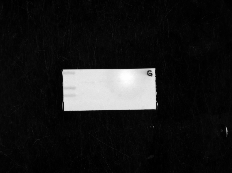


GAPDH

E-cadherin

Figure S6E

Snail

N-cadherin


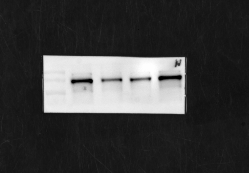

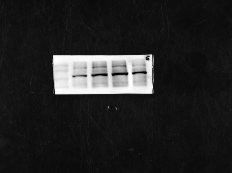

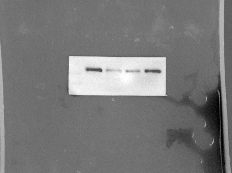

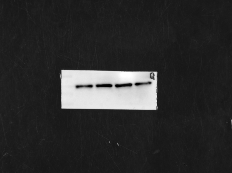


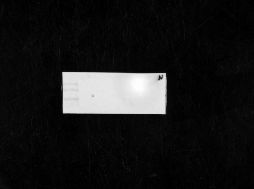

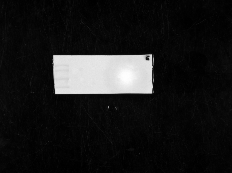

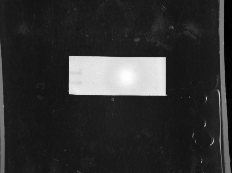

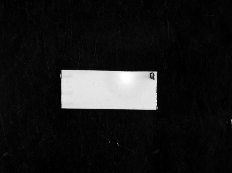


Figure S7B

GAPDH

KLLN

SMAD3


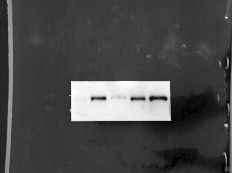

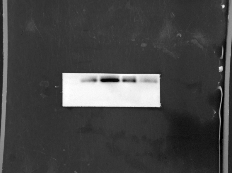

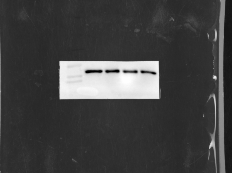


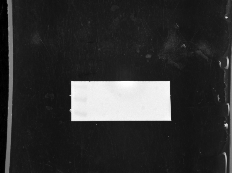

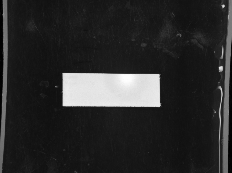

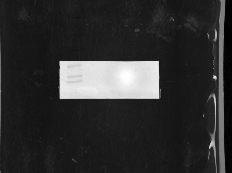


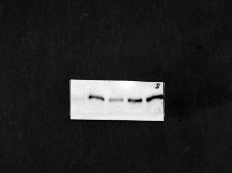

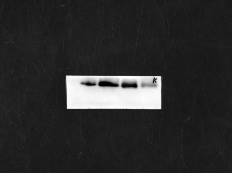

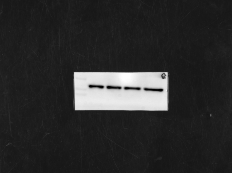


GAPDH

KLLN

SMAD3


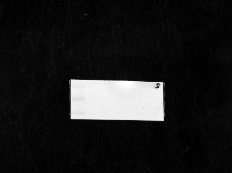

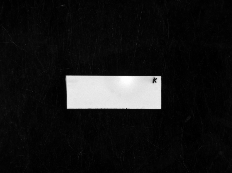

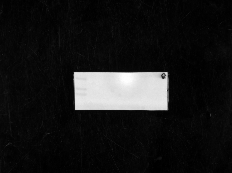

Supplement: Supplementary file 15 — Original Data File [file 41419_2022_5189_MOESM15_ESM.docx]
